# Transducin (β)-like 1 X-linked receptor 1 promotes proliferation and tumorigenicity in human breast cancer via activation of beta-catenin signaling

**DOI:** 10.1186/s13058-014-0465-z

**Published:** 2014-10-24

**Authors:** Xinghua Li, Weijiang Liang, Junling Liu, Chuyong Lin, Shu Wu, Libing Song, Zhongyu Yuan

**Affiliations:** 1 Department of Experimental Research, State Key Laboratory of Oncology in Southern China, Cancer Center, Sun Yat-sen University, Guangzhou, Guangdong China; 2 Department of Oncology, Yantai Affiliated Hospital of Binzhou Medical University, Yantai, Shandong China; 3 Department of Oncology, Nanfang Hospital, Southern Medical University, Guangzhou, Guangdong China; 4 Medicine Oncology, Cancer Center, Sun Yat-sen University, Guangzhou, Guangdong China

## Abstract

**Introduction:**

Transducin (β)-like 1 X-linked receptor 1(TBLR1) is an F-box-like and WD repeat-containing protein which functions as a switch in transcriptional activation, However, the clinical significance and biological role of TBLR1 in breast cancer remains largely unknown.

**Methods:**

Western blotting, immunocytochemistry and real-time PCR were used to evaluate TBLR1 expression in normal breast epithelial cells and breast cancer cell lines, clinical tissue samples and adjacent nontumor tissues, and in 214 paraffin-embedded specimens. Statistical analyses were used to test for the prognostic and diagnostic associations. The biological role of TBLR1 -induced proliferation and tumorigenicity in breast cancer cells was explored *in vitro* and *in vivo*. The effect of TBLR1 on the expression of cyclin D1 and β-catenin signaling was examined by Western blotting, luciferase reporter assay and by several immunoprecipitation techniques.

**Results:**

TBLR1 was significantly upregulated in breast cancer cells and tissues compared to normal control samples. Immunohistochemical analysis revealed high expression of TBLR1 in 113 of 214 (52.8%) paraffin-embedded archival breast cancer. The overall expression level of TBLR1 was significantly correlated with clinical stage (*P <*0.001), the tumor classification (*P <*0.001), node classification (*P* =0.024), and metastasis classification (*P* = 0.004), histological grade (*P* = 0.044), as well as with the expression level of c-erbB2 (*P* = 0.036) and Ki-67 (*P <*0.001). Patients with higher TBLR1 expression had shorter overall survival time, whereas patients with lower TBLR1 expression had better survival. Multivariate analysis suggested that TBLR1 expression might be an independent prognostic indicator for the survival of breast cancer patients. TBLR1 overexpression promoted, whereas TBLR1 silencing inhibited, proliferation and tumorigenicity in breast cancer cells both *in vitro* and *in vivo*. We found that TBLR1 expression was implicated in the upregulation of cyclin D1, phosphorylation of cell-cycle control protein Rb (pRb) and activation of β-catenin signaling in breast cancer.

**Conclusions:**

TBLR1 plays a key role in the development and progression of breast cancer cells via cyclin D1-transactivation and activation of the β-catenin signaling pathway. TBLR1 may be a novel prognostic marker and a potential therapeutic target in the treatment human breast cancer.

**Electronic supplementary material:**

The online version of this article (doi:10.1186/s13058-014-0465-z) contains supplementary material, which is available to authorized users.

## Introduction

Breast cancer is the most common cancer and a leading cause of cancer death among women, with approximately 232,340 new cases and 39,620 deaths predicted in the US in 2013 [1]. Although previous research has shown that the etiology of breast cancer includes heritable factors and genetic mutations [2]-[4], the molecular mechanism is poorly understood [5]. Patients with different molecular and clinical characteristics present different biological and therapeutic responses, however conventional clinical characteristics, such as tumor size, lymph node involvement, distant metastasis status and tumor differentiation, have limitations due to the complexity of tumor progression [6]-[9]. Therefore, identifying gene and protein markers will facilitate the development of novel diagnostic and prognostic indicators for improved therapies and individualized treatments in patients with breast cancer.

Transducin (β)-like 1 X-linked receptor (TBLR1) is an F-box/WD-40-containing factor which was initially identified as a component of an SMRT/N-CoR corepressor complex. It is a member of the TBL1 family, which is encoded by a distinct gene located on Chromosome 3 and is highly homologous to the TBL1 protein encoded by the TBL1X gene. TBLR1 is thought to control the switch from gene repression to gene activation of nuclear receptors and other molecules required for transcriptional activation, including estrogen receptor (ER), androgen receptor (AR), thyroid hormone receptor β (TRβ), peroxisome proliferator-activated receptor γ (PPARγ), nuclear factor kappa-light-chain-enhancer of activated B cells (NF-κb), Notch and β-catenin [10]-[16]. TBLR1 also functions as an E3-ligase by recruiting UbcH5 ubiquitin conjugating enzymes/19S proteasome, leading to the replacement of corepressors with coactivators in a ligand-dependent manner [10],[12],[16]. Although previous studies have shown that TBLR1 is highly expressed in lung squamous cell carcinoma [17], the clinical significance and biological function of TBLR1 in the progression of breast cancer remain to be established.

In this study, we found that TBLR1 was significantly upregulated in breast cancer cells and tissues both *in vitro* and *in vivo*, and that its expression was closely correlated with clinicopathologic features in breast cancer. Furthermore, we have demonstrated that TBLR1 may promote proliferation and tumorigenicity in breast cancer through cyclin D1-transactivation and activation of the β-catenin signaling pathway.

## Methods

### Cell lines

Primary normal breast epithelial cells (NBECs) were collected from a woman's mammoplasty material at the Department of Plastic Surgery, the First Affiliated Hospital of Sun Yat-sen University (PR China), according to the rules and regulations relating ethical issues on research use of human subjects in China, and established according to the previous report [18], Breast cancer cell lines, including Bcap-37 was obtained from the Cell Bank of the Chinese Academy of Sciences (Shanghai, China), MCF-7, BT-549, SKBR3, MDA-MB-231,ZR-75-1, MDA-MB-468, MDA-MB-415, MDA-MB-361, ZR-75-30,T47D and MDA-MB-435 were purchased from ATCC and cultured in Dulbecco’s modified Eagle’s medium (Gibco, Grand Island, NY, USA) supplemented with 10% fetal bovine serum (HyClone, Logan, UT, USA) and 100 μg/μL streptomycin, and 100 μg/μL penicillin in a humidified incubator containing 5% CO_2_ at 37°C.

### Patient information and tissue specimens

This study was conducted on a total of 214 paraffin-embedded breast cancer samples, which were histopathologically and clinically diagnosed at the Sun Yat-sen University Cancer Center from 1999 to 2001. Clinical and clinicopathological classification and stage were determined according to the American Joint Committee on Cancer (AJCC) criteria. The histological grade was determined according to the Elston-Ellis modification of the Scarff-Bloom-Richardson (SBR) system. Tissues were obtained from patients who were subsequently diagnosed with breast cancer: 8 freshly collected paired breast tissues and 10 freshly prepared human breast cancer tissues were frozen and stored in liquid nitrogen until further use. For the use of these clinical materials for research purposes, prior consent of the patients and approval from the Institutional Research Ethics Committee of Sun Yat-sen University were obtained. Clinical information related to the samples is summarized in Table 1.

**Table 1:** **Clinicopathological characteristics of patient samples and expression of transducin (β)-like 1 X-linked receptor 1 in breast cancer**

	Number of cases (%)
**Gender**	
Male	0 (0.0)
Female	214 (100.0)
**Age, years**	
≤47	101 (47.2)
>47	113 (52.8)
**Clinical stage**	
I	21 (9.8)
II	107 (50.0)
III	66 (30.8)
IV	20 (9.3)
**Tumor (T) classification**	
T_1_	69 (32.3)
T_2_	113 (52.8)
T_3_	24 (11.2)
T_4_	8 (3.7)
**Node (N) classification**	
N_0_	67 (31.3)
N_1_	80 (37.4)
N_2_	63 (29.4)
N_3_	4 (1.9)
**Metastasis (M) classification**	
No	192 (89.7)
Yes	22 (10.3)
**Vital status at follow up**	
Alive	146 (68.2)
Dead	68 (31.8)
**Histological differentiation**	
Well-differentiated	8 (3.7)
Moderately differentiated	152 (71.0)
Poorly differentiated	54 (25.3)
**Expression of transducin (β)-like 1 X-linked receptor 1**	
Low expression	103 (48.1)
High expression	111 (51.9)
Detectable	214 (100.0)
Undetectable	0 (0.0)
**Expression of estrogen receptor**	
0	91 (42.6)
1	98 (45.8)
2	14 (6.5)
3	11 (5.1)
**Expression of progesterone receptor**	
0	76 (35.5)
1	99 (46.3)
2	27 (12.6)
3	11 (5.1)
4	1 (0.5)
**Expression of c-erbB2**	
0	36 (16.8)
1	33 (15.4)
2	29 (13.6)
3	14 (6.5)
4	3 (1.4)

### RNA extraction, reverse transcription, and real-time PCR

Total RNA from cultured cells and fresh tissues were extracted using the Trizol reagent (Invitrogen, Carlsbad, CA, USA) according to the manufacturer's instruction. The extracted RNA was pretreated with RNase-free DNase, and about 2ug RNA from each sample was used for cDNA synthesis with iScriptcDNA Synthesis Kit (Bio-Rad Laboratories, Hercules, CA, USA). cDNAs were amplified and quantified in ABI Prism 7500 Sequence Detection System (Applied Biosystems, Texas, US) by using dye SYBR Green I (Molecular Probes, Basel, Switzerland). Expression data were normalized to the geometric mean of housekeeping gene *GAPDH* to control the variability in expression levels and calculated as:

$$2^{\left[\left(Ct\;\mathrm{of}\;\mathrm{TBLR}1\right)-\left(Ct\;\mathrm{of}\;\mathrm{GAPDH}\right)\right]}$$

where C_t_ represents the threshold cycle for each transcript.

### Plasmids and retroviral infection

A TBLR1 expression construct was generated by subcloning PCR-amplified full-length human TBLR1 cDNA into the pSin-EF2 vector. For depletion of TBLR1 to silence endogenous TBLR1, two short hairpin RNA (shRNA) oligonucleotides were cloned into the pSuper-retro-puro vector to generate pSuper-retro-TBLR1-RNAi(s), respectively. The human Cyclin D1 promoter luciferase plasmid was purchased from Addgene (Cambridge, MA, USA). Transfection of siRNAs or plasmids was performed using Lipofectamine 2000 reagent (Invitrogen, Carlsbad, CA) according to the manufacturer's instructions. Stable cell lines expressing TBLR1 and TBLR1-shRNA were generated via retroviral infection using HEK293T cells as previously described and selected with 0.5 μg/mL puromycin for 10 days.

### Western blotting

Western blot analysis was carried out according to standard methods as described previously [18], using anti-TBLR1 (Sigma, Saint Louis, MI, USA), anti-cyclin D1, anti-pRb, anti-Rb (Cell Signaling, Danvers, MA, USA). To control sample loading, the blotting membranes were stripped and re-probed with an anti-α-tubulin antibody (Sigma).

### Immunohistochemistry (IHC)

IHC staining was carried out using Histostain-Plus Kits (Invitrogen) following the manufacturer's protocols; IHC-stained tumor sections were examined and scored independently by two observers for positively stained tumor cells and the intensity of IHC signals [19]-[21]. The proportion of tumor cells was scored as follows: 0 (no positive tumor cells), 1 (1% to 25% positive tumor cells), 2 (26% to 0% positive tumor cells), 3 (51% to 75% positive tumor cells), and 4 (76% to 100% positive tumor cells). The intensity of staining was graded according to the following criteria: 0 (no staining); 1 (weak staining = light yellow), 2 (moderate staining = yellow brown), and 3 (strong staining = brown). The staining index was calculated as staining intensity score × proportion of positive tumor cells (range from 0 to 12). Optimal cutoff values were chosen on the basis of a measure of heterogeneity with the log-rank test statistical analysis with respect to overall survival.

### MTT assay

Cells were seeded in 96-well plates at an initial density of 2 × 10^3^/well. At each time point, cells were stained with 100 μL sterile 3-(4,5-Dimethyl-2-thiazolyl)- 2,5-diphenyl-2H-tetrazolium bromide (MTT) dye (0.5 mg/mL, Sigma) for 4 hours at 37°C, followed by removal of the culture medium and addition of 100 μL of dimethyl sulphoxide (Sigma). The absorbance was measured at 570 nm, with 655 nm as the reference wavelength. All experiments were carried out in triplicates.

### Anchorage-independent growth ability assay

Five hundred cells were trypsinized and suspended in 2 mL of complete medium plus 0.3% agar (Sigma). The agar-cell mixture was plated on top of a bottom layer with 1% agar completed medium mixture. At 10 days, viable colonies that were larger than 0.1 mm were counted. The experiment was carried out for each cell line in triplicate.

### Colony formation assays

Cells were plated in 6-well plates (1 × 10^3^ cells per plate) and cultured for 10 days. The colonies were stained with 1% crystal violet for 30 seconds after fixation with 4% formaldehyde for 5 minutes.

### Flow cytometry

Cells were harvested, washed with cold 1 × PBS, and processed for cell cycle analysis using flow cytometry. Briefly, the cells were fixed in 75% ethanol and stored at 4°C overnight for later analysis. The fixed cells were centrifuged at 1,000 rpm for 5 minutes and washed with cold PBS twice. RNase A (20 μg/ml final concentration) and propidium iodide staining solution (50 μg/ml final concentration) were added to the cells and incubated for 30 minutes at 37°C in the dark. Twenty thousand cells were analyzed using a CytomicsTM FC 500 instrument (Beckman Coulter, Brea, California, USA) equipped with CXP software. Modfit LT 3.1 trial cell cycle analysis software was used to determine the percentage of cells in the different phases of the cell cycle.

### Bromodeoxyuridine labeling and immunofluorescence

Cells (5 × 10^4^) were plated on coverslips (Fisher 12-545-80, Fisher Scientific, Pittsburgh, PA, USA). After 24s, cells were incubated with 5-bromodeoxyuridine (BrdU) for 1 hour and stained with anti-BrdU antibody (Upstate, Billerica, MA, USA) according to the manufacturer's instruction. Gray-level images were acquired under a laser scanning microscope (Axioskop 2 plus; Carl Zeiss Co Ltd, Oberkochen, Germany).

### Xenografted tumor model

Female BALB/c-nu mice (4-5 weeks of age, 14 to 16 g) were purchased from the Center of Experimental Animal of Guangzhou University of Chinese Medicine, and were housed in barrier facilities on a 12-hour light/dark cycle. All experimental procedures were approved by the Institutional Animal Care and Use Committee of Sun Yat-sen University. The BALB/c nude mice were randomly divided into twp groups (n =5/group). A 0.72 mg E2 60-day release pellet (Innovative Research of America) was implanted subcutaneously on the dorsal side of each mouse 1 day before tumor cell implantation to support the growth of the estrogen-dependent MCF-7 cell-derived tumors. For tumor cell implantation, MCF-7-TBLR1 or MCF-7-TBLR1-RNAi or their respective control cells (1 × 10^7^) in 100 μL of the mixture were subcutaneously injected into the mice. Tumors were examined once every other day: length, width, and thickness measurements were obtained with calipers and tumor volumes were calculated. On day 42, animals were euthanized, and tumors were excised and weighed.

### Luciferase reporter assay

Cells (3 × 10^3^) were seeded in triplicate in 48-well plates and allowed to settle for 24 hours, then 100 ng luciferase reporter plasmids or the control luciferase plasmid plus 1 ng pRL-TK renilla plasmid (Promega, Madison, Wisconsin) were transfected into breast cancer cells using Lipofectamine 2000 reagent (Invitrogen). Forty-eight hours after transfection, Dual-Luciferase Reporter Assay (Promega) was performed according to the manufacturer's instructions. Three independent experiments were performed and the data are presented as the mean ± SD.

### Chromatin immunoprecipitation (ChIP) assays

Cells (2 × 10^6^) were plated per 100-mm diameter dish and treated with formaldehyde to crosslink chromatin-associated proteins to DNA. The cells were trypsinized and resuspended in lysis buffer, and nuclei were isolated and sonicated to shear the DNA to 500 bp to 1 kb fragments (verified by agarose gel electrophoresis). Equal aliquots of chromatin supernatants were separated and incubated with different antibodies as indicated or anti-IgG antibody as a negative control overnight at 4°C with rotation. DNA was extracted and the promoters of cyclin D1 and MYC were amplified by PCR. All ChIP assays were performed three times and representative results are presented.

### Statistical analysis

All statistical analyses were carried out using the SPSS version 16.0 statistical software packages. The correlation between TBLR1 expression and the clinicopathological characteristics was analyzed by the chi-square test. Survival curves were plotted by the Kaplan-Meier method and compared with the log-rank test. The significance of various variables for survival was analyzed by the Cox proportional hazards model in the multivariate analysis. The differences between experimental conditions were compared using Student's *t*-test. *P <*0.05 was considered statistically significant.

## Results

### TBLRis upregulated in human breast cancer cells

**Figure 1:**
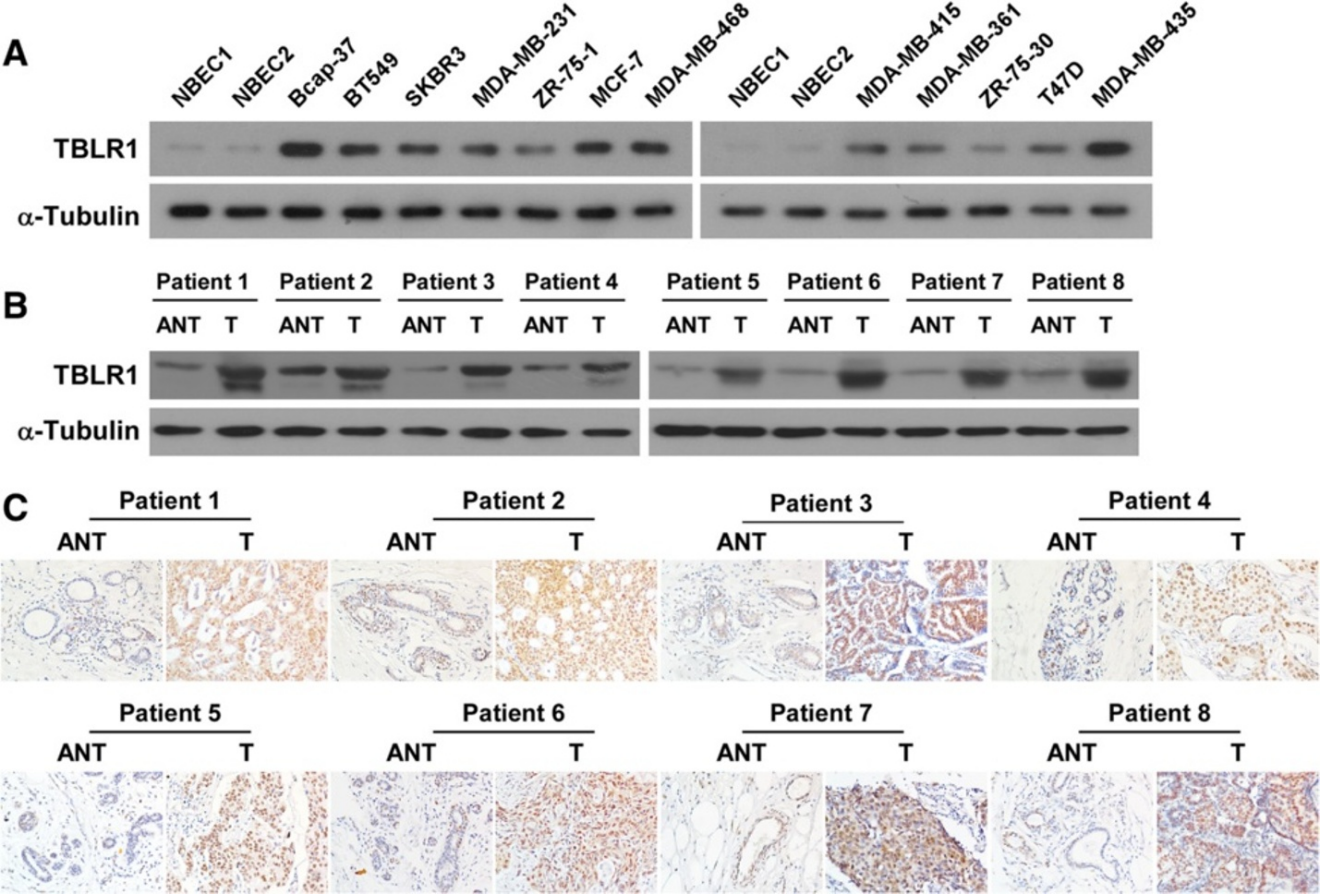
**Transducin (β)-like 1 X-linked receptor 1 (TBLR1) expression is elevated in breast cancer cells and tissues. (A)** Western blotting shows the TBLR1 protein levels in normal breast epithelial cells (NBEC) and in 12 breast cancer cell lines; α-Tubulin was used as a loading control. **(B and C)** Western blotting and immunohistochemistry staining, respectively, show the expression levels of TBLR1 protein in each of the primary breast tumor tissue samples (T) and adjacent non-tumor tissues (ANT) from the same patient.

Expression of TBLR1 was examined in breast cancer cells and tissues. The results of western blot and real-time PCR analyses showed that expression of TBLR1 was significantly upregulated at both the protein and mRNA levels in 12 tested breast cancer cell lines relative to NBECs (Figure 1A; Additional file 1: Figure S1A). Similarly, western blotting and IHC showed that TBLR1 protein was upregulated in breast cancer tissue samples compared to their matched adjacent non-tumor tissues from the same patient (Figure 1B and C); and real-time PCR revealed that TBLR1 mRNA was also significantly upregulated in breast cancer tissues (Additional file 1: Figure S1B).

### TBLRexpression is associated with clinical features in breast cancer

**Figure 2:**
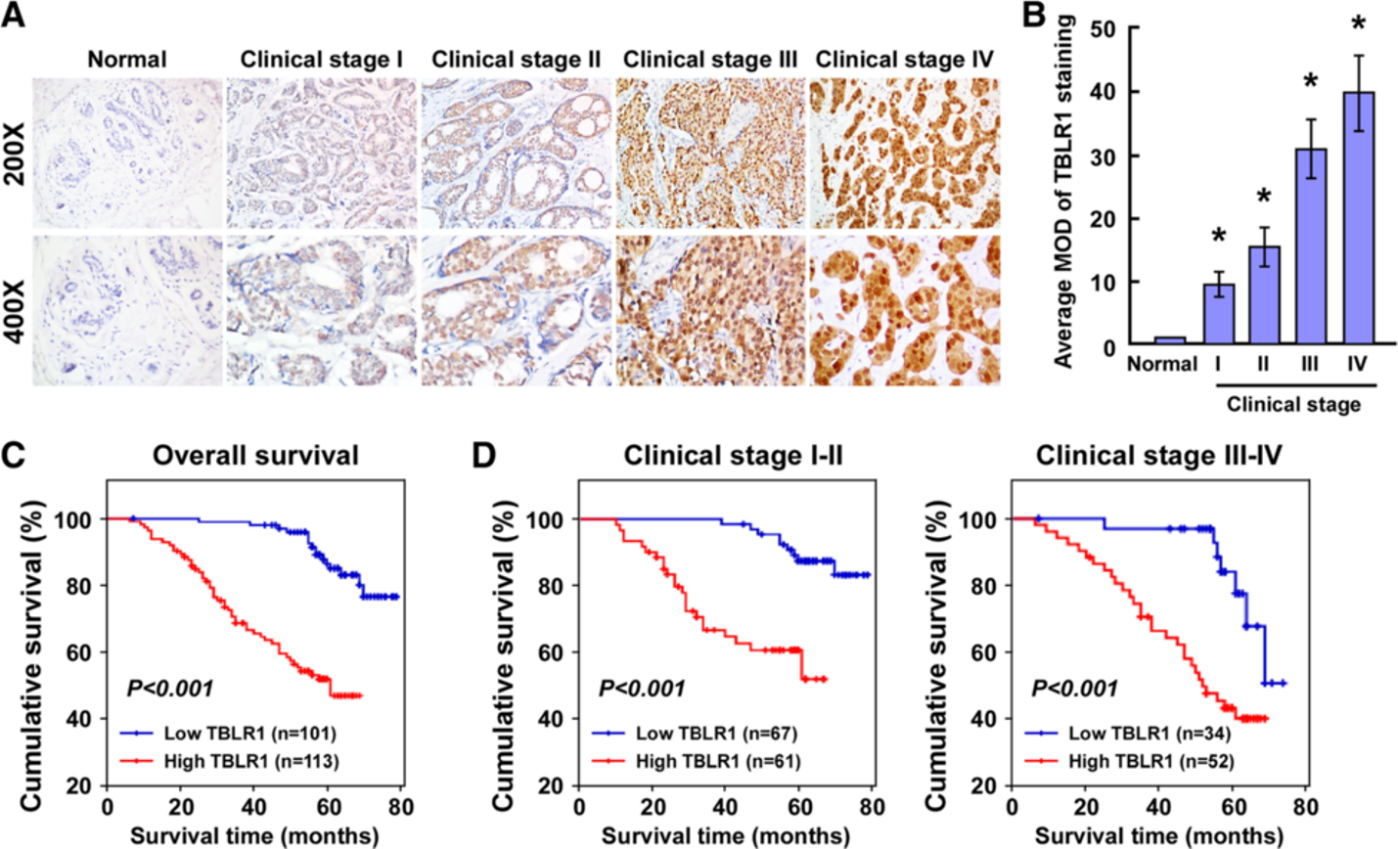
**Transducin (β)-like 1 X-linked receptor 1 (TBLR1) is upregulated in archived breast cancer tissues. (A)** Representative immunohistochemistry fluorescent micrographs showing TBLR1 expression levels in normal and tumor breast tissue samples from cases with different clinical stages (I to IV); magnification, 200× and 400×. **(B)** Quantification analyses of mean TBLR1 expression levels in normal breast tissues (n = 3) and breast tumor tissues from cases with different clinical stages (n =16/stage); MOD, mean optical density. **(C)** Kaplan-Meier survival curves comparing cumulative overall survival rates in patients with low and high TBLR1 expression levels. **(D)** Survival curves for breast cancer patients with clinical stage I to II (left) and clinical stage III to IV (right) disease.

The clinical significance of the upregulation of TBLR1 in breast cancer was examined by IHC and correlated with the clinical characteristics of 214 paraffin-embedded breast cancer tissue specimens, including 21 (9.8%) cases with stage I, 107 (50.0%) cases with stage II, 66 (30.8%) cases with stage III and 20 (9.3%) cases with stage IV tumors. We found that TBLR1 was markedly upregulated in breast cancer tissues, but was only detectable at low levels in normal breast tissues (Figure 2A). Quantitative analysis indicated that the average mean optical density (MOD) of TBLR1 staining in clinical stage I to IV primary tumors were statistically significantly higher than the value in normal breast tissues (*P <*0*.*001; Figure 2B). As shown in Table 2, detailed analyses revealed that expression of TBLR1was significantly associated with clinical stage (*P* <0.001), tumor (T) classification (*P* <0.001), node (N) classification (*P* = 0.024), metastasis (M) classification (*P* = 0.004), pathological differentiation (*P* = 0.044), c-erbB-2 expression (*P =* 0.036) and Ki-67 expression (*P* <0.001). However, it was not associated with patient age, estrogen receptor (ER) or progesterone receptor (PR) status. The Spearman correlation coefficients between TBLR1 expression and clinical stage, T classification, N classification, M classification, pathological differentiation, and Ki-67 expression were 0.215 (*P* = 0.002), 0.522 (*P* <0.001), 0.139 (*P* = 0.042), 0.197 (*P* = 0.004), 0.136 (*P* = 0.047), and 0.630 (*P* <0.001), respectively (Table 3). Taken together, these results indicated that expression of TBLR1 was correlated with many of the key clinical features of breast cancer.

**Table 2:** **Clinicopathological characteristics of patient samples and expression of TBLR1 in breast cancer and correlation between TBLR1 expression and clinicopathological characteristics of breast cancer patients**

Characteristics		Total	Transducin (β)-like 1 X-linked receptor 1 (TBLR1)		Chi-square test***P***-value
			Low expression (%)	High expression (%)	
Age, years	<47	101	53 (50.0)	48 (44.4)	0.416
	≥47	113	53 (50.0)	60 (55.6)	
Clinical stage	I	21	17 (16.8)	4 (3.5)	<0.001
	I	107	50 (49.5)	57 (50.4)	
	III	66	31 (30.7)	35 (31.0)	
	IV	20	3 (3.0)	17 (15.0)	
Tumor classification	T_1_	69	57 (56.4)	12 (10.6)	<0.001
	T_2_	113	41 (40.6)	72 (63.7)	
	T_3_	24	3 (3.0)	21 (18.6)	
	T_4_	8	0 (0.0)	8 (7.1)	
Node classification	N_0_	67	40 (39.6)	27 (23.9)	0.024
	N_1_	80	32 (31.7)	48 (42.5)	
	N_2_	63	29 (28.7)	34 (30.1)	
	N_3_	4	0 (0.0)	4 (3.5)	
Metastasis classification	Yes	22	4 (4.0)	18 (16.0)	0.004
	No	192	97 (96.0)	95 (84.1)	
Pathological differentiation	Well-differentiated	8	3 (3.0)	5 (4.4)	0.044
	Moderately differentiated	152	80 (79.2)	72 (63.7)	
	Poorly differentiated	54	18 (17.8)	36 (31.9)	
Expression of estrogen receptor	0	91	47 (46.5)	44 (38.9)	0.545
	1	98	41 (40.6)	57 (50.4)	
	2	14	7 (6.9)	7 (6.2)	
	3	11	6 (5.9)	5 (4.4)	
Expression of progesterone receptor	0	76	33 (32.7)	43 (38.1)	0.139
	1	99	53 (52.5)	46 (40.7)	
	2	27	12 (11.9)	15 (13.3)	
	3	11	2 (2.0)	9 (8.0)	
	4	1	1 (1.0)	0 (0.0)	
Expression of C-erbB-2	0	36	16 (26.2)	20 (37.0)	0.036
	1	33	25 (41.0)	8 (14.8)	
	2	29	12 (20.0)	17 (31.5)	
	3	14	6 (9.8)	8 (14.8)	
	4	3	2 (3.3)	1 (1.9)	
Expression of Ki-67	1	111	86 (85.1)	25 (22.1)	<0.001
	2	103	15 (14.9)	88 (77.9)	

**Table 3:** **Spearman correlation between transducin (β)-like 1 X-linked receptor 1 (TBLR1) and clinical pathologic factors**

Variables	TBLR1 expression level	
	Spearman correlation	***P***-value
Clinical staging	0.215	0.002
Tumor classification	0.522	<0.001
Node classification	0.139	0.042
Metastasis classification	0.197	0.004
Pathological differentiation	0.136	0.047
Expression of Ki67	0.630	<0.001
Survival time	−0.512	<0.001

### Increased expression of TBLRis correlated with the prognosis of breast cancer patients

Patient survival analysis was conducted and revealed that TBLR1 protein expression in primary breast cancer was significantly inversely correlated with the survival time of patients (*r* = 0.512, *P* <0.001; Table 3). Kaplan-Meier survival curves showed that patients with high levels of TBLR1 had significantly shorter overall survival (OS) rates than those with low levels of TBLR1 (*P* <0.001; Figure 2C). The cumulative 5-year survival rates in patients with low levels of TBLR1 expression were 86.4% (95% confidence interval 0.791 to 0.937), compared to 51.9% (95% confidence interval 0.421 to 0.617) in those with high levels of TBLR1 expression. Furthermore, univariate and multivariate analyses confirmed that clinical stage, pathological differentiation and Ki-67, as well as TBLR1 expression, were identified as independent prognostic factors, as shown in Table 4. Taken together, these results indicated that TBLR1 might be a novel and potentially valuable independent prognostic biomarker in patients with breast cancer. The prognostic value of TBLR1 expression in patients with breast cancer was also evaluated by analyzing survival times in different patient subgroups according to clinical stage. We found that the patients with high TBLR1 expression had significantly lower OS rates compared with those with a low level of TBLR1 expression in the early clinical subgroup (stages I to II, n = 128; log-rank, *P* <0.001; Figure 2D, left panel) and the advanced disease subgroup (stages III to IV, n = 86; log-rank, *P* <0.001; Figure 2D, right panel). All in all, our data suggest that TBLR1 might be a novel and potentially useful independent biomarker for the prognosis of patients with breast cancer.

**Table 4:** **Univariate and multivariate analyses of various prognostic parameters in patients with breast cancer Cox-regression analysis**

	Univariate analysis			Multivariate analysis		
	Patients, number	***P***-value	Regression coefficient (SE)	***P***-value	Relative risk	95% confidence interval
Transducin (β)-like 1 X-linked receptor 1		<0.001	1.642 (0.301)	0.012	2.590	1.237, 5.422
Low expression	103					
High expression	111					
**Ki67**		<0.001	1.518 (0.283)	0.034	2.126	1.058, 4.271
Low expression	111					
High expression	103					
**Clinical stage**						
I	21					
II	107	<0.001	0.804 (0.158)	0.007	1.593	1.135, 2.237
III	66					
IV	20					
**Pathological differentiation**		0.001	0.873 (0.258)	0.026	1.809	1.074, 3.050
Well-differentiated	8					
Moderately differentiated	154					
Poorly differentiated	52					

Transducin (β)-like 1 X-linked receptor 1 (TBLR1) protein expression level in breast cancer significantly correlated with patient survival time (*P* <0.001). The correlation coefficient was -0.512 indicating that higher levels of TBLR1 expression correlate with shorter survival time. For 5-year survival: low expression, 86.4% (79.1% to 93.7%); high expression, 51.9% (42.1% to 61.7%).

### TBLRpromotes proliferation in breast cancer cells

The biological role of TBLR1 in breast cancer was further explored by employing IHC to examine the relationship between TBLR1 and Ki-67 in breast cancer tissues (Tables 2, 3 and Additional file 2: Figure S2). The results supported our earlier findings by showing that TBLR1 was positively correlated with Ki-67 expression. This suggests that upregulation of TBLR1 promoted proliferation in breast cancer cells.

**Figure 3:**
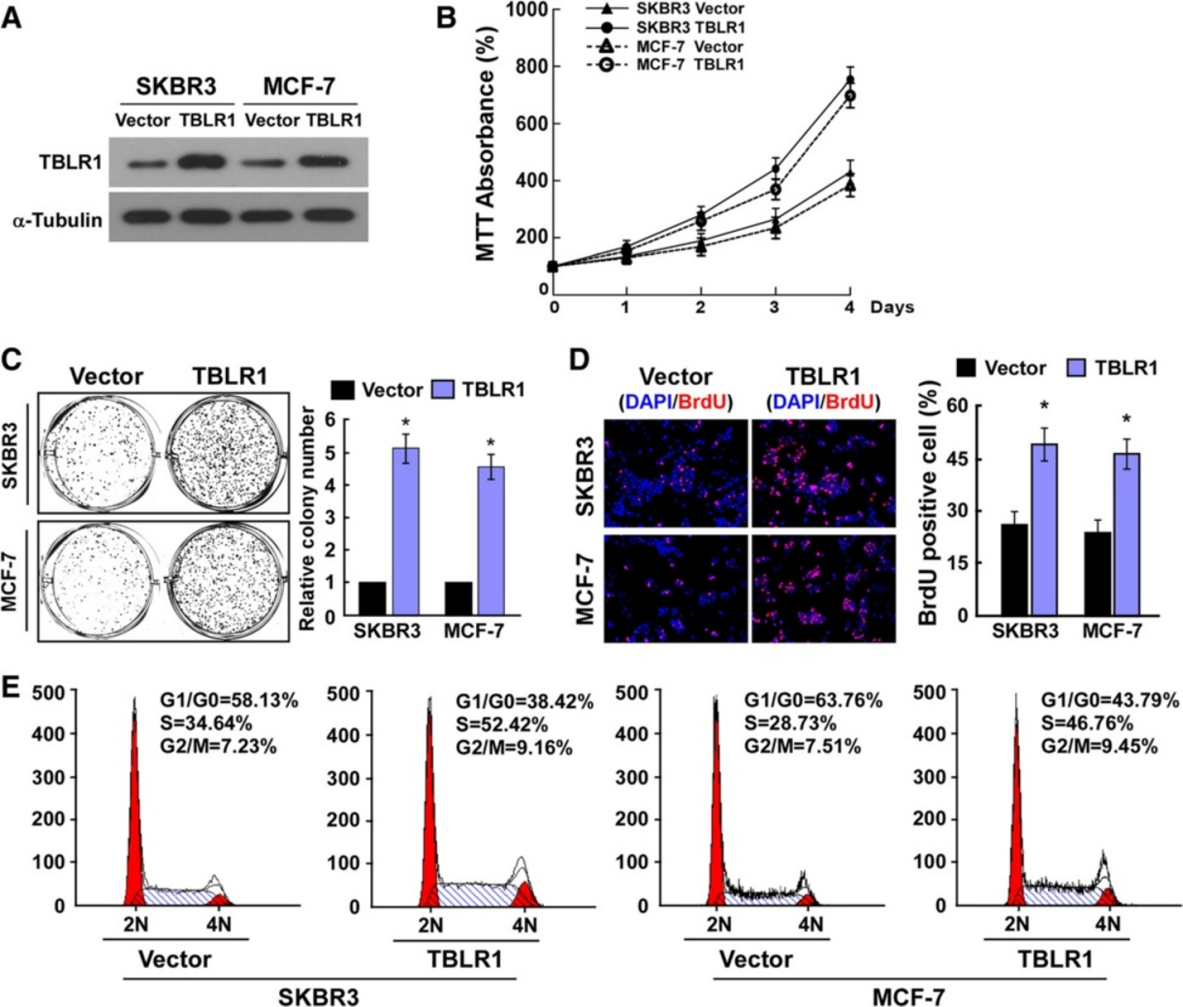
**Transducin (β)-like 1 X-linked receptor 1 (TBLR1) overexpression promotes cell proliferation and G1-S phase transition in breast cancer cells. (A)** Western blotting shows the levels of TBLR1 protein in TBLR1-overexpressing breast cancer cell lines; glyceraldehyde-3-phosphate dehydrogenase was used as a loading control. **(B and C)** MTT and colony formation assays, respectively, show that growth rates and colony sizes are increased in TBLR1-overexpressing breast cancer cells. **(D)** Representative micrographs (left panel) and quantification (right panel) of 5-bromodeoxy uridine (BrdU)-incorporated breast cancer cells. DAPI, 4',6-diamidino-2-phenylindole. **(E)** Flow cytometric analyses showing the percentages of cells of TBLR1-overexpressing cells and vector control cells at different phases of the cell-cycle. The data are representative of three independent experiments. All values are given as the mean ± SD of three independent experiments; *P <0.05.

**Figure 4:**
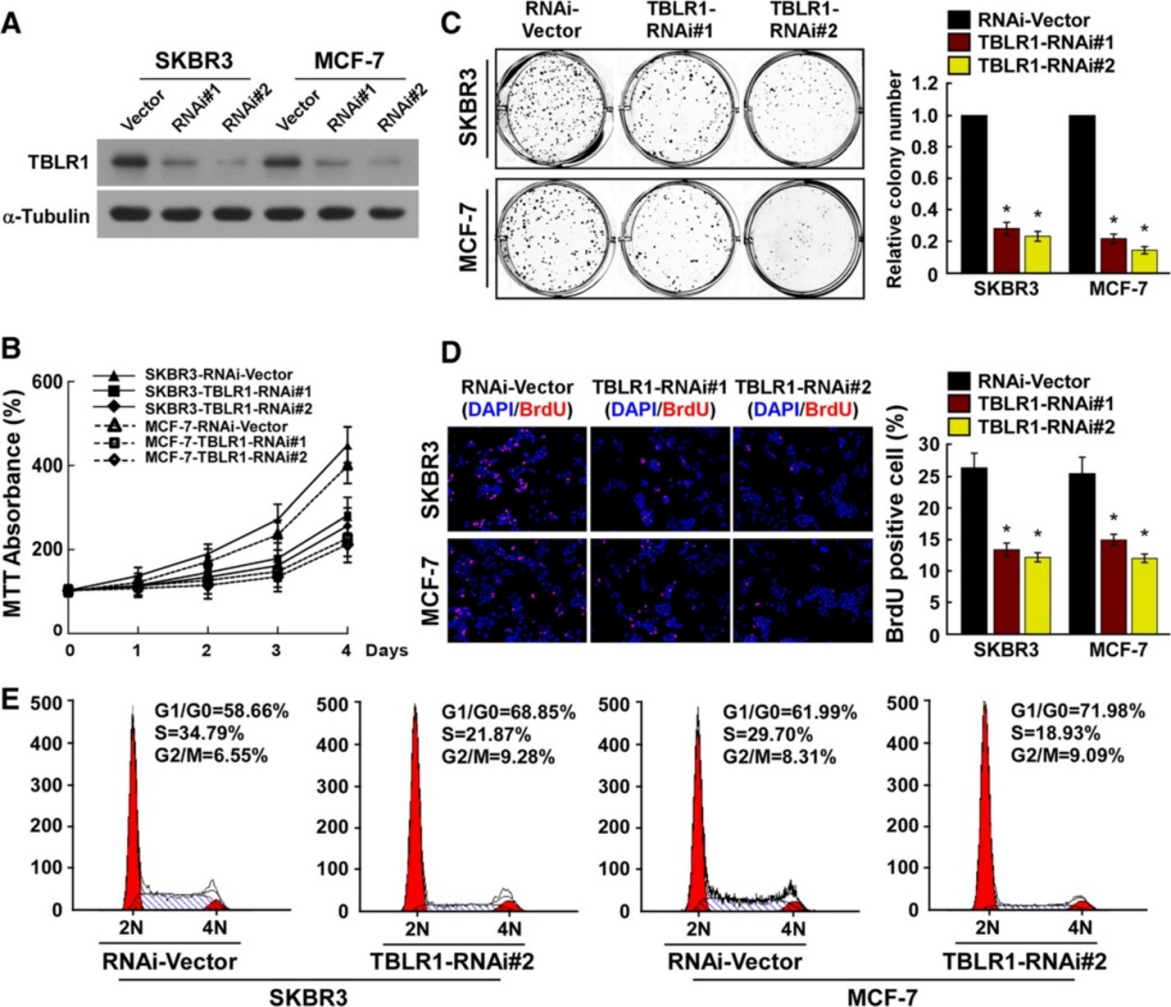
**Transducin (β)-like 1 X-linked receptor 1 (TBLR1) silencing inhibits cell proliferation and G1-S phase transition in breast cancer cells. (A)** Western blotting shows the levels of TBLR1 protein in TBLR1-silenced breast cancer cells; glyceraldehyde-3-phosphate dehydrogenase was used as a loading control. **(B and C)** MTT and colony formation assays, respectively, show that growth rates and colony sizes are decreased in TBLR1-silenced cells. DAPI, 4',6-diamidino-2-phenylindole. **(D)** Representative micrographs (left panel) and quantification (right panel) of 5-bromodeoxy uridine (BrdU)-incorporated breast cancer cells. **(E)** Flow cytometric analyses showing the percentages of TBLR1-shRNA transfected cells and vector controls at different phases of the cell cycle. The data are representative of three independent experiments. All values are given as the mean ± SD of three independent experiments; *P <0.05.

To confirm the biological role of TBLR1 in breast cancer, stable cell lines overexpressing TBLR1 were established by subcloning full-length human TBLR1 cDNA into the pSin-EF2 vector (Figure 3A). MTT assays showed an approximately two-fold increase in the number of TBLR1-overexpressing cells relative to vector control cells after four days of culture (Figure 3B), indicating that ectopic expression of TBLR1 increased the proliferative capacity of breast cancer cells. A similar result was shown by the colony formation assays (Figure 3C). Conversely, knockdown of endogenous TBLR1 expression using two TBLR1-specific shRNAs (Figure 4A) showed that TBLR1-silencing significantly inhibited cell proliferation, leading to more than a two-fold decrease in cell number compared to vector controls after 4 days of culture (*P* <0.05; Figure 4B). These results were consistent with the colony formation assays (Figure 4C). A BrdU-incorporation assay was performed in SKBR3 and MCF-7 cell lines to assess the mechanism underlying the promotion of cellular proliferation by TBLR1. The results showed that the percentage of BrdU positive cells was significantly increased in TBLR1-overexpressing cells and was significantly decreased in TBLR1-knockdown cells (Figures 3D and 4D). Similarly, flow cytometric analysis showed that overexpression of TBLR1 markedly decreased the percentage of cells in G0/G1 phase and increased the percentage of cells in S phase; whereas silencing TBLR1 induced the converse effects (Figures 3E and 4E). In line with our earlier observations, these results confirmed that TBLR1 is involved in promoting proliferation in breast cancer cells.

### TBLRpromotes the tumorigenicity of breast cancer cells in vitro and in vivo

**Figure 5:**
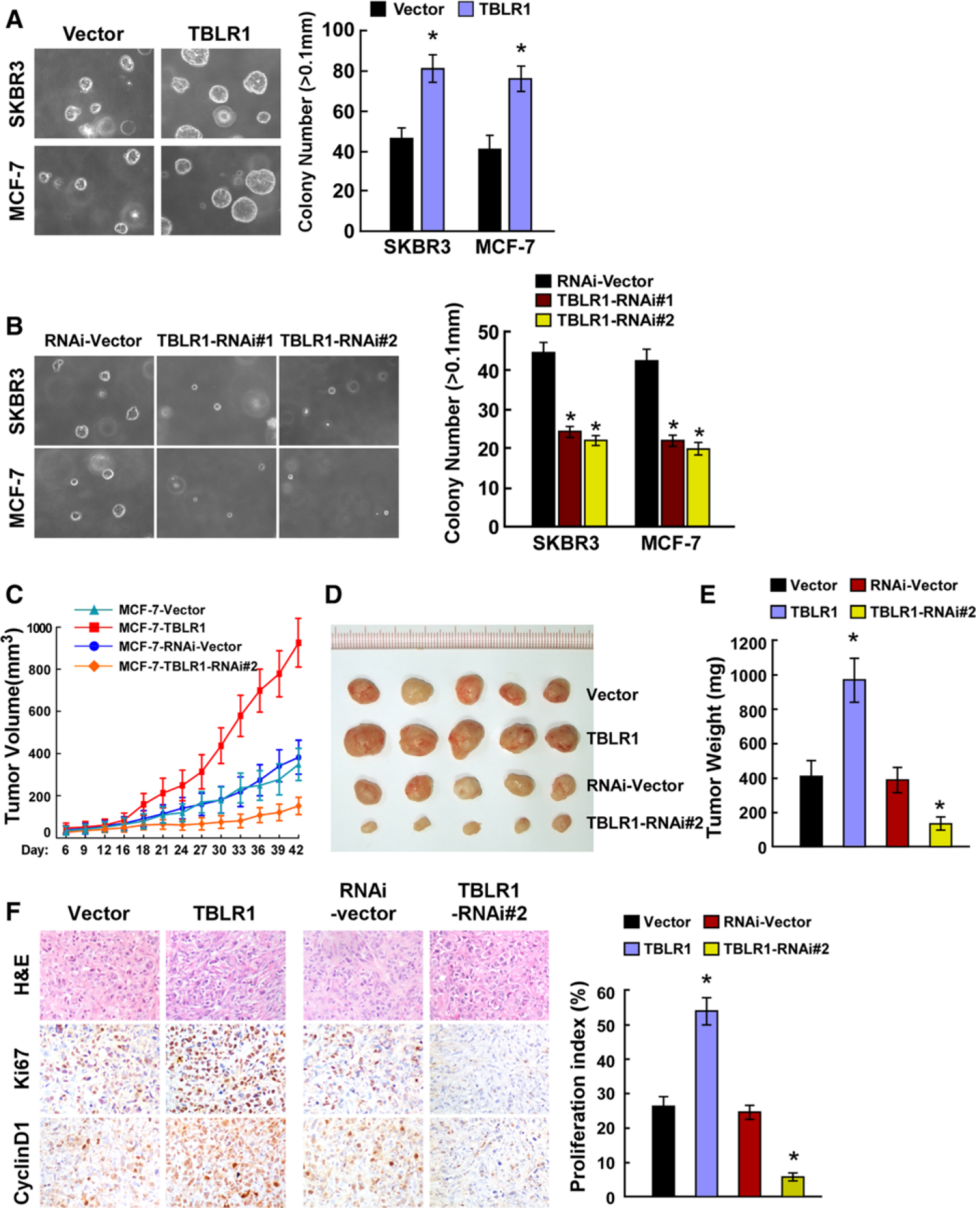
**Transducin (β)-like 1 X-linked receptor 1 (TBLR1) contributes to breast cancer progression** ***in vitro and in vivo*** **.** **(A and B)** Anchorage-independent growth assays in TBLR1-overexpressing cells and TBLR1-silenced cells, respectively. Quantification of the soft agar colony formations was based on colonies >0.1 mm in diameter after 10 days of culture. **(C-E)** Xenograft model in nude mice inoculated with either TBLR1-overexpressing or TBLR1-knockdown breast cancer cells, or their respective vector control cells (n =5/group) 42 days after inoculation. **(C)** Growth curves showing changes in tumor volume; **(D)** images showing changes in tumor size; **(E)** mean tumor weights. **(F)** H&E and immunohistochemical staining showed that overexpression of TBLR1 induced, whereas suppression of TBLR1 inhibited, the tumorigenicity of breast cancer cells *in vivo*, as indicated by the percentage of Ki-67 positive cells. All data are presented as mean ± SD of three independent experiments; *P <0.05.

Because TBLR1 expression was correlated with the clinical staging and T classification of breast cancer (Tables 2 and 3), we further evaluated the effect of TBLR1 on the tumorigenic activity of breast cancer cells. Anchorage-independent growth assays demonstrated that the growth capacity of SKBR3 and MCF-7 breast cancer cell lines was significantly enhanced by TBLR1-overexpression, as shown by the increased number and size of colonies (Figure 5A). Conversely, the growth capacity of both SKBR3 and MCF-7 cells could be compromised by downregulation of TBLR1, as revealed by the decreased number and size of colonies (Figure 5B). Moreover, to determine whether TBLR1 promoted tumorigenicity in breast cancer cells *in vivo*, we established a xenograft model by inoculating nude mice (n =5/group) with MCF-7/TBLR1 or MCF-7/TBLR1-RNAi cells or their respective control vectors (MCF-7/vector or MCF-7/RNAi-vector). The tumors formed by the TBLR1-overexpressing cells grew significantly faster, were larger and had greater masses than those formed by their vector controls at each time point; whereas, tumors formed by TBLR1-knockdown cells grew significantly slower, were smaller and had lower masses than those formed by shRNA vector control cells (*P* <0.001; Figure 5C,D and E). Of note, IHC revealed that tumors formed by TBLR1-overexpressing cells exhibited higher levels of cyclin D1 and Ki-67 staining, and a greater proliferative index than tumors formed by TBLR1-knockdown cells (Figure 5F). Therefore, the *in vivo* results were consistent with our *in vitro* observations, confirming that TBLR1 plays an important role in enhancing the tumorigenicity of breast cancer cells.

### TBLRpromotes cell proliferation via activation of the β-catenin signaling pathway

**Figure 6:**
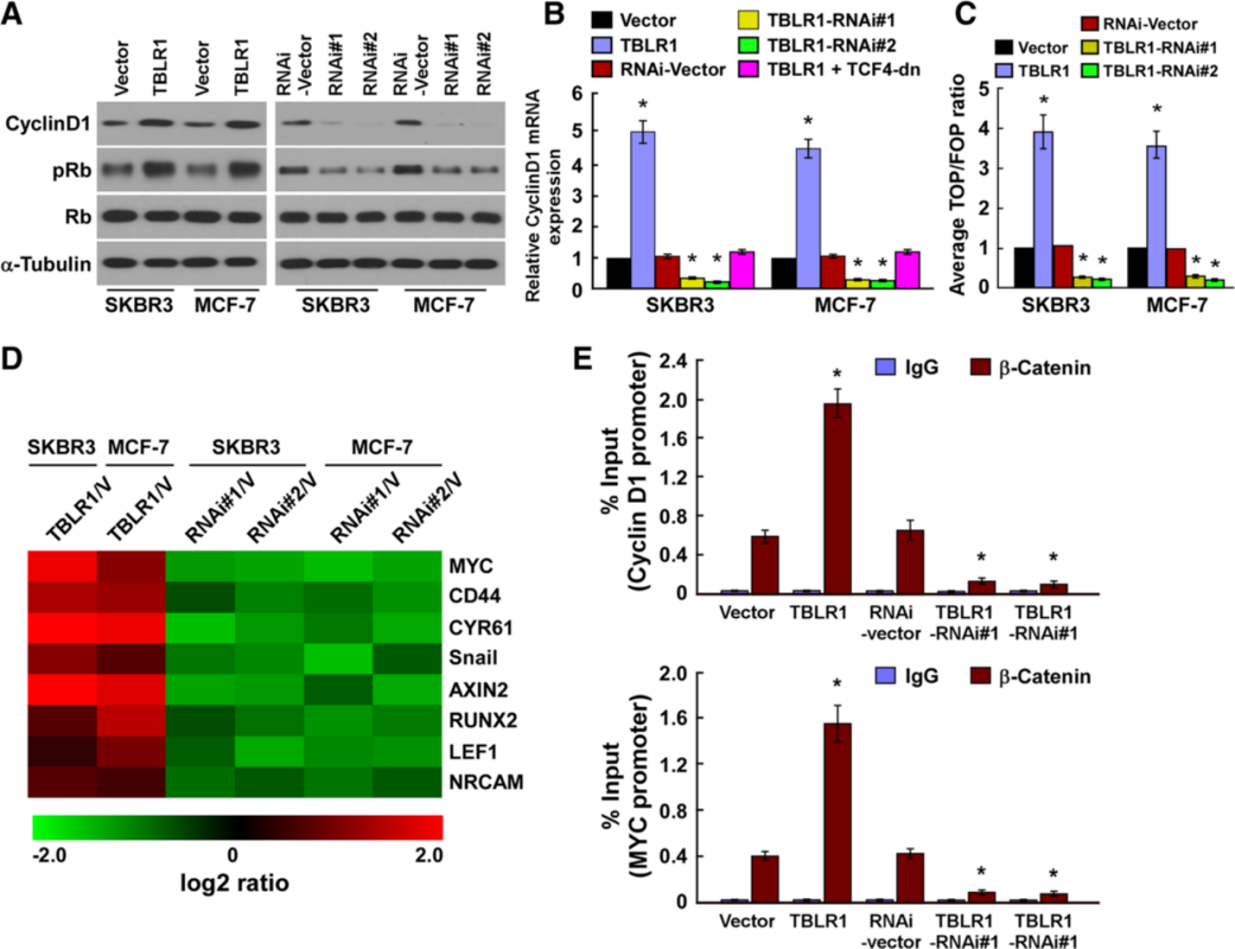
**Transducin (β)-like 1 X-linked receptor 1 (TBLR1) activates β-catenin signaling. (A)** Western blotting shows the protein levels of cyclin D1, pRb and total Rb in breast cancer cell lines; α-tubulin was used as a loading control. **(B)** Real-time PCR analysis shows the relative expression of cyclin D1 mRNA in breast cancer cell lines. Expression levels were normalized against glyceraldehyde-3-phosphate dehydrogenase. **(C)** Indicated cells transfected with TOPflash or FOPflash and Renilla pRL-TK plasmids were subjected to dual-luciferase assays 48 hours after transfection. Reporter activity detected was normalized by Renilla luciferase activity. **(D)** Real-time PCR analysis indicates an apparent overlap between β-catenin-dependent genes and TBLR1-regulated genes. The pseudocolor represents the intensity scale of TBLR1 versus vector control, or TBLR1 siRNA versus negative control, generated by a log2 transformation. **(E)** Chromatin immunoprecipitation assays showing the levels of endogenous β-catenin protein bound to *cyclin D1* and *MYC* promoters in TBLR1 breast cancer cells and vector control cells; Error bars represents the mean ± SD of three independent experiments; *P <0.05.

Consistent with the aforementioned results, the phosphorylated Rb was also increased in the TBLR1 overexpressing-cells and decreased in the TBLR1 silencing cells, further demonstrating that TBLR1 plays an important role in the proliferation of HCC cells (Figure 6A). Interestingly, we found that both transcriptional and translational levels of CDK regulator cyclin D1 were upregulated by TBLR1 overexpression, but supressed by TBLR1 silencing, as compared with that in control cells (Figure 6A and B).

Importantly, the stimulatory effect of TBLR1 on the cyclin D1 expression was abolished by the inhibition of Wnt/β-catenin signaling via TCF4 dominant-negative mutant (TCF4-dn) transfection, suggesting that TBLR1 might activate Wnt/β-catenin signaling. As expected, the β-catenin/TCF4 activity and the expression levels of eight classically recognized Wnt/β-catenin target genes were significantly increased in the TBLR1 overexpressing breast cancer cells, but decreased in the TBLR1 silencing cells (Figure 6C and D, Additional file 3: Figure S3). Moreover, the ChIP assay and Cyclin D1 promoter activity assay revealed that TBLR1 might upregulate *cyclin D1* and *MYC* expression via direct binding to their promoters (Figure 6E and Additional file 4: Figure S4).

### Wnt/β-catenin signaling activation is vital to TBLR1-induced cell proliferation

**Figure 7:**
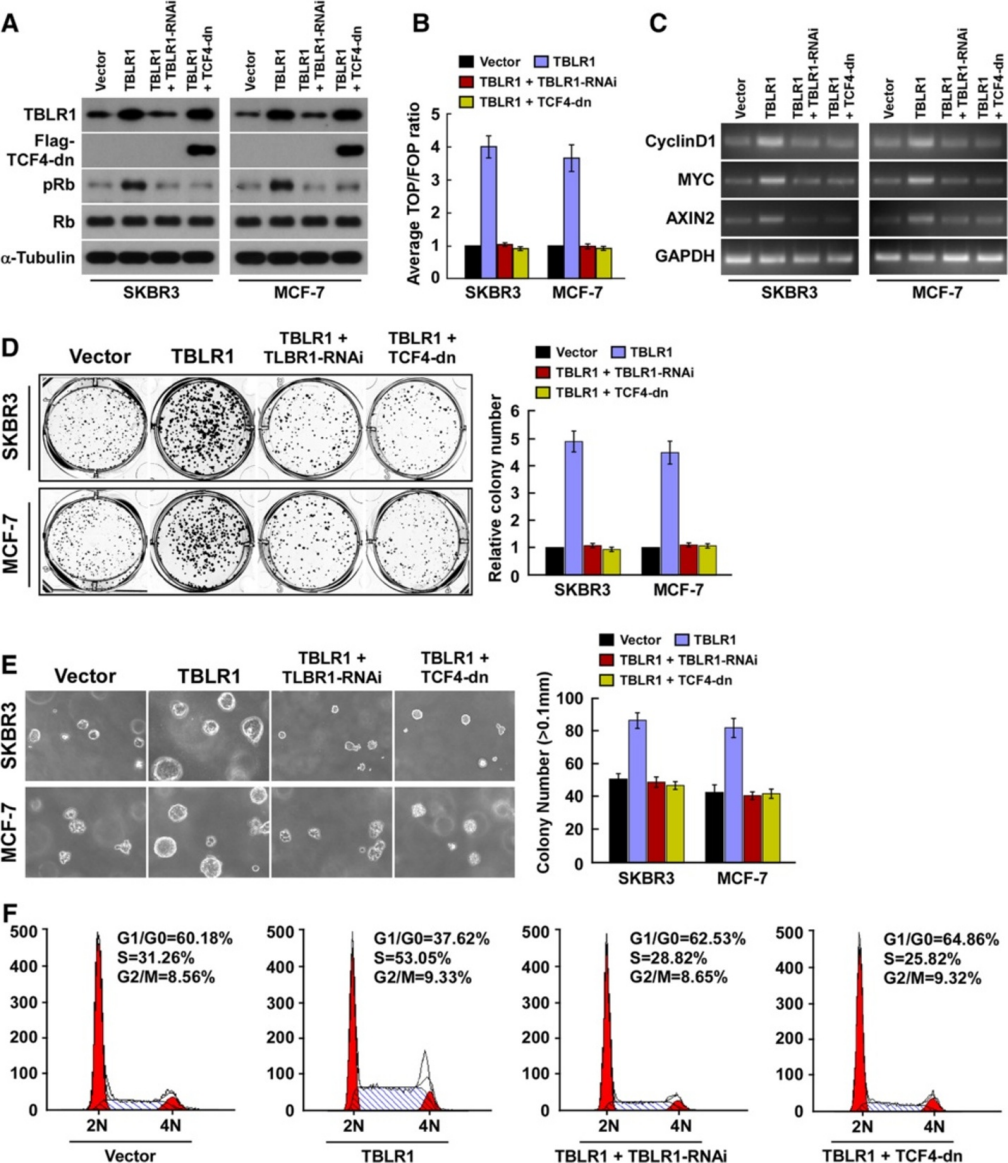
**Inhibition of Wnt/β-catenin signaling blocked the functional role of transducin (β)-like 1 X-linked receptor 1 (TBLR1). (A)** Western blotting shows the protein levels of TBLR1, TCF4-dn, pRb and total Rb in breast cancer cell lines; β-tubulin was used as a loading control. **(B)** Indicated cells transfected with TOPflash or FOPflash and Renilla pRL-TK plasmids were subjected to dual-luciferase assays 48 hours after transfection. Reporter activity detected was normalized by Renilla luciferase activity. **(C)** PCR analysis shows the expression of cyclin D1, MYC and AXIN2 mRNA in breast cancer cell lines. Expression levels were normalized to glyceraldehyde-3-phosphate dehydrogenase. **(D)** Representative micrographs (left) and quantification (right) of crystal violet-stained cell colonies. **(E)** Anchorage-independent growth assays in indicated cells. **(F)** Flow cytometry analysis examined the portions of cell cycle in SKBR3 cells.

We further examined whether inhibiting wnt/β-catenin signaling has a similar effect to knocking down TBLR1 expression. As shown in Figure 7A-C, knocking down of TBLR1 or blocking wnt/β-catenin signaling by ectopically expressing TCF4-dn, drastically reduced Rb phosphorylation, TOP/FOP activity and transcription of Cyclin D1, MYC and AXIN2 in TBLR1-overexpressing cells. Moreover, both knocking down of TBLR1 and inhibition of wnt/β-catenin signaling, abrogated the stimulatory effect of TBLR1 overexpression on breast cancer cell growth and proliferation (Additional file 5: Figure S5 and Figure 7D-F). Thus, our results indicated that functional wnt/β-catenin signaling activation is vital to TBLR1-induced proliferation of breast cancer cells.

### TBLR1 expression is positively correlated with cyclin D1 and β-catenin in human breast cancer tissues

**Figure 8:**
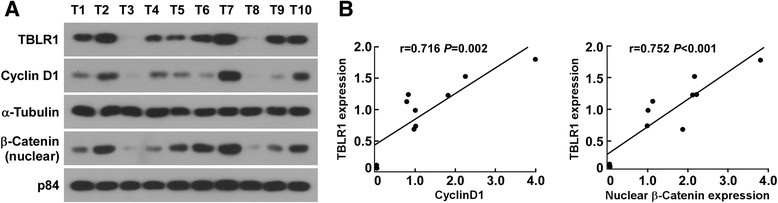
**Transducin (β)-like 1 X-linked receptor 1 (TBLR1) expression is positively correlated with cyclin D1 and nuclear β-catenin expression in breast cancer tissues.** **(A and B)** Western blotting (left) and correlation analyses (right) of TBLR1, Cyclin D1 and nuclear β-catenin in 10 freshly prepared human breast cancer tissues; α-Tubulin and P84 were used as the loading controls.

To confirm whether the results obtained in breast cancer cell lines could also be observed in human primary breast tumors, we examined the expression of TBLR1, cyclin D1 and β-catenin in 10 freshly prepared breast cancer tissues. Western blot and correlation analyses showed that expression of TBLR1 was positively correlated with expression of cyclin D1 (*r* = 0.716; *P* = 0.002) and nuclear β-catenin (*r* = 0.752; *P* <0.001) (Figure 8A and B). These results further demonstrated that TBLR1 mediated proliferation in breast cancer cells *via* activation of the β-catenin signaling pathway.

## Discussion

The pivotal finding of this study is that the elevated expression of TBLR1 correlated with a poor prognosis and reduced survival of breast cancer patients, suggesting that TBLR1 is a potential independent prognostic factor for breast cancer. We found that TBLR1 is upregulated in breast cancer cells at both the mRNA and protein levels compared with normal breast epithelial cells. Overexpression of TBLR1 promotes the proliferation and tumorigenicity of breast cancer cells *in vitro* and *in vivo*. Moreover, we showed that overexpression TBLR1 activated wnt/β-catenin signaling pathway. These findings provide strong evidence that upregulation of TBLR1 plays an important role in promoting progression of breast cancer.

Tumorigenesis is a complex multi-step process characterized by uncontrolled cell growth and tumor formation. Reports have shown that tumorigenesis is associated with the progressive accumulation of genetic and epigenetic alterations in genes and proteins that regulate cell proliferation, cell death and genomic instability [22],[23]. Therefore, identifying molecules that promote tumorigenesis is central to the development of novel diagnostic and prognostic markers, and potential therapeutic targets in cancer. In this study, we found that upregulation of TBLR1 dramatically promoted cell proliferation and tumorigenicity in breast cancer cells, both *in vitro* and *in vivo*. Furthermore, expression of TBLR1 was significantly correlated with clinicopathological characteristics and patient survival in breast cancer. Importantly, knocking down of TBLR1 markedly reduced breast cancer cell proliferation and tumorigenicity. Moreover, TBLR1 upregulation was also observed in lung squamous cell carcinoma [17], suggeting that TBLR1 upregulation might be a common event in the progression of different cancers. Thus, our findings uncovered the oncogenic function of TBLR1 in the development and progression of breast cancer, and suggested TBLR1 might be a novel prognostic marker and therapeutic target.

Of note, we found that TBLR1 expression was positively correlated with the expression of C-erbB-2, which has been found to be frequently amplified or overexpressed, to play an important role in the development and progression of certain aggressive types of breast cancer [24],[25]. Functioning as a transcription co-factor, TBLR1 has been reported to transcriptionally upregulate MYC and AXIN2 expression via binding to the β-catenin/TCF4 transcription complex [13]. Thus, it would be of great interest and importance to investigate whether TBLR1 upregulates C-erbB-2 expression via β-catenin/TCF4 transcription complex or others, and whether knocking down of TBLR1 could reduce the tumorigencity of C-erbB-2-overexpressing breast tumors.

It has been reported that cyclin D1 plays an important role in tumor development through regulation of the cell cycle [26]-[29]. Our results showed that expression of TBLR1 in human breast cancer cells was positively correlated with expression of cyclin D1 and nuclear β-catenin, and activation of genes downstream of β-catenin. This indicates that TBLR1 may induce proliferation and tumorigenesis in breast cancer *via* activation of the β-catenin signaling pathway. Our findings have provided new insights into the pathogenesis of breast cancer, and indicate that TBLR1 might be a potential therapeutic target in breast cancer.

## Conclusion

We have shown that TBLR1 expression is elevated in breast cancer cells and tissues. The elevated expression of TBLR1 is significantly correlated with the progression of breast cancer. TBLR1 overexpression promoted, whereas TBLR1 silencing inhibited, proliferation and tumorigenicity in breast cancer cells both *in vitro* and *in vivo*. Furthermore, we have demonstrated that TBLR1 may promote proliferation and tumorigenicity in breast cancer cells *via* activation of the β-catenin signaling pathway, which provides a novel molecular mechanism for breast cancer progression. However, further investigations are required to elucidate the precise role of TBLR1 in the progression of breast cancer. Our results suggest that TBLR1 may be an effective clinical marker of disease progression and a prognostic indicator of survival in patients with breast cancer.

## Authors' contributions

XL participated in study design and performed the statistical analysis, carried out the majority of the practical experimentation and drafted the whole manuscript. WL and JL assisted with *in vitro* experiments and most of the *in vivo* experiments. CL contributed to experimental design and the animal research. SW assisted in western blotting, PCR experiments and immunochemistry staining and participated in patient survival analysis. LS provided assistance in study design and experimental interpretation. ZY conceived of the study, and participated in its design and coordination and helped to draft the manuscript. All authors contributed to revise the manuscript and approved the final version for publication.

## Additional files

## Electronic supplementary material

Additional file 1: Figure S1.: Expression of transducin (β)-like 1 X-linked receptor 1 (TBLR1) is elevated in breast cancer. **(A)** Expression analyses of TBLR1 mRNA in primary normal breast epithelial cells (NBECs) and cultured breast cancer cell lines by real-time PCR. Glyceraldehyde-3-phosphate dehydrogenase was used as a loading control. **(B)** Real-time PCR analysis showing expression of TBLR1 mRNA in each of the primary breast cancer tissue samples (T) and adjacent noncancerous tissues (ANT) from the same patient. Error bars represents the mean ± SD of three independent experiments; *P <0.05. (TIFF 125 KB)

Additional file 2: Figure S2.: Quantitative analysis of transducin (β)-like 1 X-linked receptor 1 (TBLR1) and Ki-67 in human breast cancer samples by immunohistochemical staining. Quantification indicates the relationship between TBLR1 and Ki-67 expression (*P* <0.001); 1 = low expression; 2 = high expression. (TIFF 37 KB)

Additional file 3: Figure S3.: Transducin (β)-like 1 X-linked receptor 1 (TBLR1) increased AXIN2 expression. Real-time PCR analysis of AXIN2 expression in TBLR1-overexpressing, TBLR1-silencing, and control cells. Glyceraldehyde-3-phosphate dehydrogenase was used as a loading control. Error bars represents the mean ± SD of three independent experiments; *P <0.05. (TIFF 48 KB)

Additional file 4: Figure S4.: Transducin (β)-like 1 X-linked receptor 1 (TBLR1) increased the luciferase activity of Cyclin D1 promoter. Indicated cells transfected with Cyclin D1 promoter luciferase and Renilla pRL-TK plasmids were subjected to dual-luciferase assays 48 hours after transfection. Reporter activity detected was normalized by Renilla luciferase activity. Error bars represents the mean ± SD of three independent experiments; *P <0.05. (TIFF 44 KB)

Additional file 5: Figure S5.: Inhibition of Wnt/β-catenin signaling blocked the functional role of transducin (β)-like 1 X-linked receptor 1 (TBLR1). **(A)** MTT assays showed the proliferation rate of indicated cells. **(B)** Representative micrographs (left) and quantification of 5-bromodeoxy uridine (BrdU)-positive cells. Error bars represents the mean ± SD of three independent experiments; *P <0.05. (TIFF 589 KB)

Below are the links to the authors’ original submitted files for images.

Authors’ original file for figure 1

Authors’ original file for figure 2

Authors’ original file for figure 3

Authors’ original file for figure 4

Authors’ original file for figure 5

Authors’ original file for figure 6

Authors’ original file for figure 7

Authors’ original file for figure 8

## Abbreviations

AJCC: American Joint Committee on Cancer
AR: androgen receptor
bp: base pairs
BrdU: 5-bromodeoxy uridine
ChIP: chromatin immunoprecipitation
DAPI: 4',6-diamidino-2-phenylindole
ER: estrogen receptor
GAPDH: glyceraldehyde-3-phosphate dehydrogenase
H&E: hematoxylin and eosin
IHC: immunocytochemistry
M classification: metastasis classification
MOD: mean optical density
MTT: 3-(4,5-Dimethylthiazol-2-yl)-2,5-diphenyltetrazolium bromide
N classification: node classification
NBECs: primary normal breast epithelial cells
NF-Κb: nuclear factor kappa-light-chain-enhancer of activated B cells
OS: overall survival
PBS: phosphate-linked buffer
PPARγ: peroxisome proliferator-activated receptor γ
pRb: phosphorylation of cell-cycle control protein Rb
*r*: correlation coefficient
SBR: Scarff-Bloom-Richardson
shRNA: short hairpin RNA
T classification: tumor classification
TBLR1: transducin (β)-like 1 X-linked receptor 1
TRβ: thyroid hormone receptor β

## References

[1] Desantis C, Ma J, Bryan L, Jemal A (2014). Breast cancer statistics, 2013. CA Cancer J Clin.

[2] Lichtenstein P, Holm NV, Verkasalo PK, Iliadou A, Kaprio J, Koskenvuo M, Pukkala E, Skytthe A, Hemminki K (2000). Environmental and heritable factors in the causation of cancer-analyses of cohorts of twins from Sweden, Denmark, and Finland. N Engl J Med.

[3] Knudson AG (1995). Hereditary cancers: from discovery to intervention. J Natl Cancer Inst Monogr.

[4] McPherson K, Steel CM, Dixon JM (2000). ABC of breast diseases. Breast cancer-epidemiology, risk factors, and genetics. BMJ.

[5] Ye Y, Qiu TH, Kavanaugh C, Green JE (2004). Molecular mechanisms of breast cancer progression: lessons from mouse mammary cancer models and gene expression profiling. Breast Dis.

[6] Gonzalez-Angulo AM, Morales-Vasquez F, Hortobagyi GN (2007). Overview of resistance to systemic therapy in patients with breast cancer. Adv Exp Med Biol.

[7] Elston CW, Ellis IO, Pinder SE (1999). Pathological prognostic factors in breast cancer. Crit Rev Oncol Hematol.

[8] Galea MH, Blamey RW, Elston CE, Ellis IO (1992). The Nottingham Prognostic Index in primary breast cancer. Breast Cancer Res Treat.

[9] Fitzgibbons PL, Page DL, Weaver D, Thor AD, Allred DC, Clark GM, Ruby SG, O'Malley F, Simpson JF, Connolly JL, Hayes DF, Edge SB, Lichter A, Schnitt SJ (2000). Prognostic factors in breast cancer. College of American Pathologists Consensus Statement 1999. Arch Pathol Lab Med.

[10] Perissi V, Aggarwal A, Glass CK, Rose DW, Rosenfeld MG (2004). A corepressor/coactivator exchange complex required for transcriptional activation by nuclear receptors and other regulated transcription factors. Cell.

[11] Yoon HG, Chan DW, Huang ZQ, Li J, Fondell JD, Qin J, Wong J (2003). Purification and functional characterization of the human N-CoR complex: the roles of HDAC3, TBL1 and TBLR1. EMBO J.

[12] Perissi V, Scafoglio C, Zhang J, Ohgi KA, Rose DW, Glass CK, Rosenfeld MG (2008). TBL1 and TBLR1 phosphorylation on regulated gene promoters overcomes dual CtBP and NCoR/SMRT transcriptional repression checkpoints. Mol Cell.

[13] Li J, Wang CY (2008). TBL1-TBLR1 and beta-catenin recruit each other to Wnt target-gene promoter for transcription activation and oncogenesis. Nat Cell Biol.

[14] Keutgens A, Shostak K, Close P, Zhang X, Hennuy B, Aussems M, Chapelle JP, Viatour P, Gothot A, Fillet M, Chariot A (2010). The repressing function of the oncoprotein BCL-3 requires CtBP, while its polyubiquitination and degradation involve the E3 ligase TBLR1. Mol Cell Biol.

[15] Choi HK, Choi KC, Yoo JY, Song M, Ko SJ, Kim CH, Ahn JH, Chun KH, Yook JI, Yoon HG (2011). Reversible SUMOylation of TBL1-TBLR1 regulates beta-catenin-mediated Wnt signaling. Mol Cell.

[16] Glass CK, Ogawa S (2006). Combinatorial roles of nuclear receptors in inflammation and immunity. Nat Rev Immunol.

[17] Liu Y, Sun W, Zhang K, Zheng H, Ma Y, Lin D, Zhang X, Feng L, Lei W, Zhang Z, Guo S, Han N, Tong W, Feng X, Gao Y, Cheng S (2007). Identification of genes differentially expressed in human primary lung squamous cell carcinoma. Lung Cancer.

[18] Li J, Zhang N, Song LB, Liao WT, Jiang LL, Gong LY, Wu J, Yuan J, Zhang HZ, Zeng MS, Li M (2008). Astrocyte elevated gene-1 is a novel prognostic marker for breast cancer progression and overall patient survival. Clin Cancer Res.

[19] Song LB, Liao WT, Mai HQ, Zhang HZ, Zhang L, Li MZ, Hou JH, Fu LW, Huang WL, Zeng YX, Zeng MS (2006). The clinical significance of twist expression in nasopharyngeal carcinoma. Cancer Lett.

[20] Geisler SA, Olshan AF, Weissler MC, Cai J, Funkhouser WK, Smith J, Vick K (2002). p16 and p53 Protein expression as prognostic indicators of survival and disease recurrence from head and neck cancer. Clin Cancer Res.

[21] Fukuoka J, Fujii T, Shih JH, Dracheva T, Meerzaman D, Player A, Hong K, Settnek S, Gupta A, Buetow K, Hewitt S, Travis WD, Jen J (2004). Chromatin remodeling factors and BRM/BRG1 expression as prognostic indicators in non-small cell lung cancer. Clin Cancer Res.

[22] Ponder BA (2001). Cancer genetics. Nature.

[23] Bartek J (2011). DNA damage response, genetic instability and cancer: from mechanistic insights to personalized treatment. Mol Oncol.

[24] Mitri Z, Constantine T, O'Regan R (2012). The HER2 Receptor in Breast Cancer: Pathophysiology, Clinical Use, and New Advances in Therapy. Chemother Res Pract.

[25] Burstein HJ (2005). The distinctive nature of HER2-positive breast cancers. N Engl J Med.

[26] Lumachi F, Luisetto G, Basso SM, Basso U, Brunello A, Camozzi V (2011). Endocrine therapy of breast cancer. Curr Med Chem.

[27] Arber N, Hibshoosh H, Moss SF, Sutter T, Zhang Y, Begg M, Wang S, Weinstein IB, Holt PR (1996). Increased expression of cyclin D1 is an early event in multistage colorectal carcinogenesis. Gastroenterology.

[28] Zhang T, Nanney LB, Luongo C, Lamps L, Heppner KJ, DuBois RN, Beauchamp RD (1997). Concurrent overexpression of cyclin D1 and cyclin-dependent kinase 4 (Cdk4) in intestinal adenomas from multiple intestinal neoplasia (Min) mice and human familial adenomatous polyposis patients. Cancer Res.

[29] Lee RJ, Albanese C, Stenger RJ, Watanabe G, Inghirami G, Haines GK, Webster M, Muller WJ, Brugge JS, Davis RJ, Pestell RG (1999). pp 60(v-src) induction of cyclin D1 requires collaborative interactions between the extracellular signal-regulated kinase, p38, and Jun kinase pathways. A role for cAMP response element-binding protein and activating transcription factor-2 in pp60(v-src) signaling in breast cancer cells. J Biol Chem.

