# Supplementary figures and images for: Transducin (β)-like 1 X-linked receptor 1 promotes proliferation and tumorigenicity in human breast cancer via activation of beta-catenin signaling
Source: Breast Cancer Res. 2014 Oct 24;16:465. doi: 10.1186/s13058-014-0465-z (PMC4303137; doi:10.1186/s13058-014-0465-z)

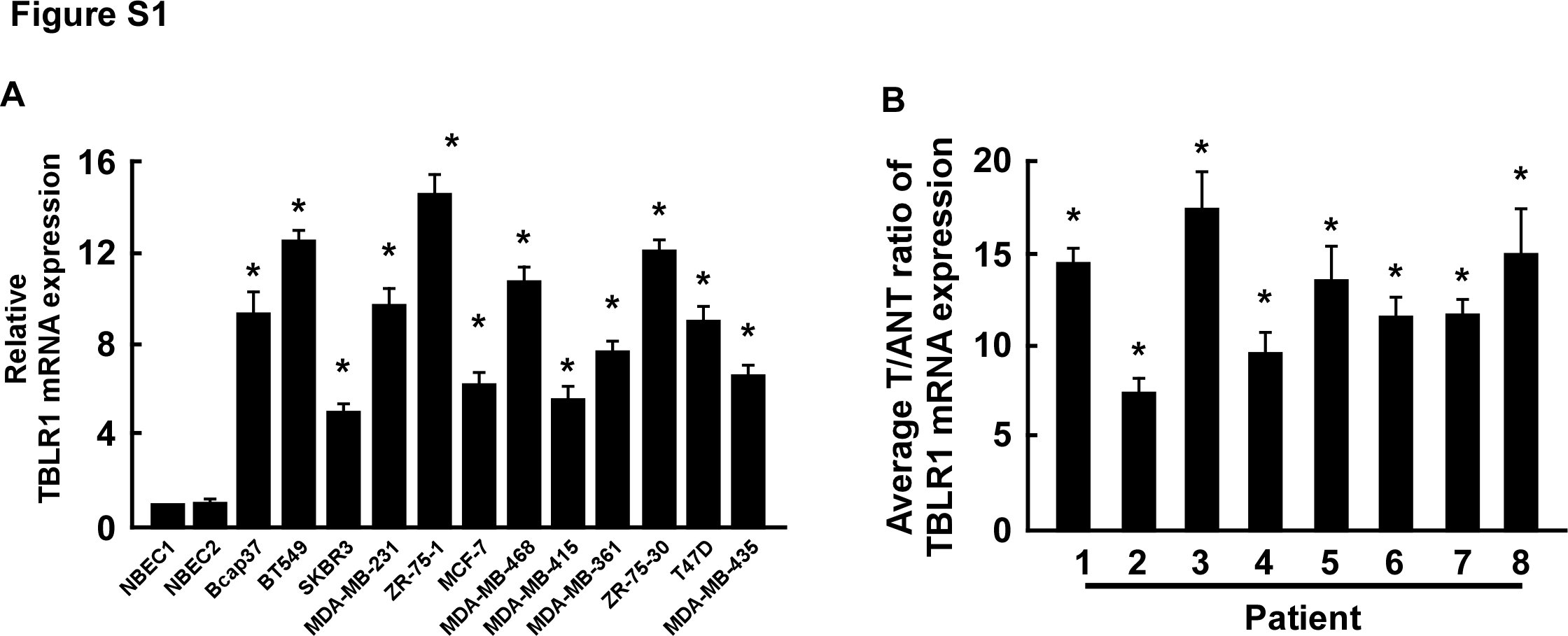

Supplement: Supplementary file 1 — Additional file 1: Figure S1.: Expression of transducin (β)-like 1 X-linked receptor 1 (TBLR1) is elevated in breast cancer. (A) Expression analyses of TBLR1 mRNA in primary normal breast epithelial cells (NBECs) and cultured breast cancer cell lines by real-time PCR. Glyceraldehyde-3-phosphate dehydrogenase was used as a loading control. (B) Real-time PCR analysis showing expression of TBLR1 mRNA in each of the primary breast cancer tissue samples (T) and adjacent noncancerous tissues (ANT) from the same patient. Error bars represents the mean ± SD of three independent experiments; *P <0.05. (TIFF 125 KB) [file 13058_2014_465_MOESM1_ESM.tiff]

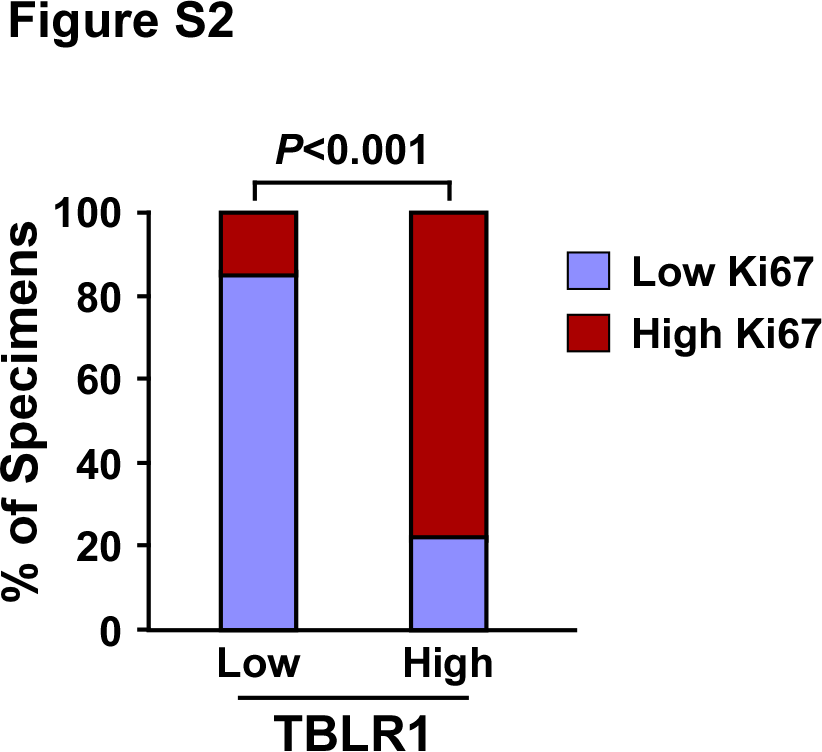

Supplement: Supplementary file 2 — Additional file 2: Figure S2.: Quantitative analysis of transducin (β)-like 1 X-linked receptor 1 (TBLR1) and Ki-67 in human breast cancer samples by immunohistochemical staining. Quantification indicates the relationship between TBLR1 and Ki-67 expression (P <0.001); 1 = low expression; 2 = high expression. (TIFF 37 KB) [file 13058_2014_465_MOESM2_ESM.tiff]

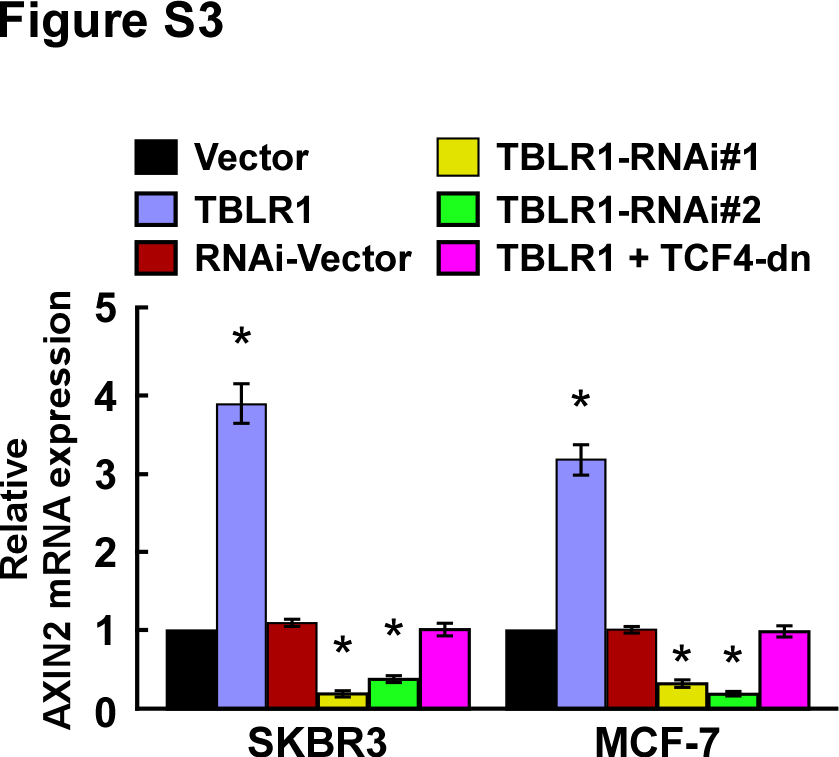

Supplement: Supplementary file 3 — Additional file 3: Figure S3.: Transducin (β)-like 1 X-linked receptor 1 (TBLR1) increased AXIN2 expression. Real-time PCR analysis of AXIN2 expression in TBLR1-overexpressing, TBLR1-silencing, and control cells. Glyceraldehyde-3-phosphate dehydrogenase was used as a loading control. Error bars represents the mean ± SD of three independent experiments; *P <0.05. (TIFF 48 KB) [file 13058_2014_465_MOESM3_ESM.tiff]

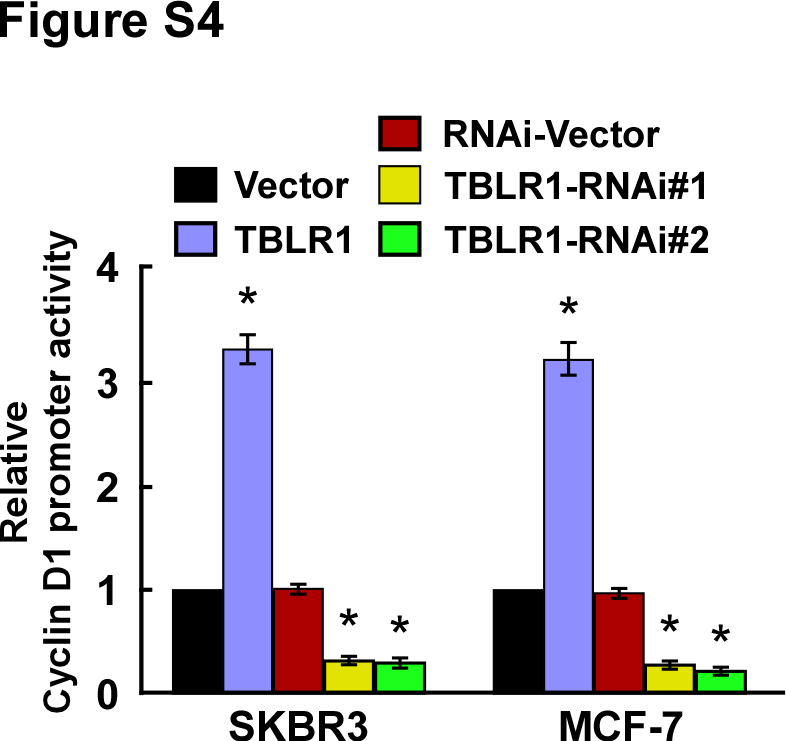

Supplement: Supplementary file 4 — Additional file 4: Figure S4.: Transducin (β)-like 1 X-linked receptor 1 (TBLR1) increased the luciferase activity of Cyclin D1 promoter. Indicated cells transfected with Cyclin D1 promoter luciferase and Renilla pRL-TK plasmids were subjected to dual-luciferase assays 48 hours after transfection. Reporter activity detected was normalized by Renilla luciferase activity. Error bars represents the mean ± SD of three independent experiments; *P <0.05. (TIFF 44 KB) [file 13058_2014_465_MOESM4_ESM.tiff]

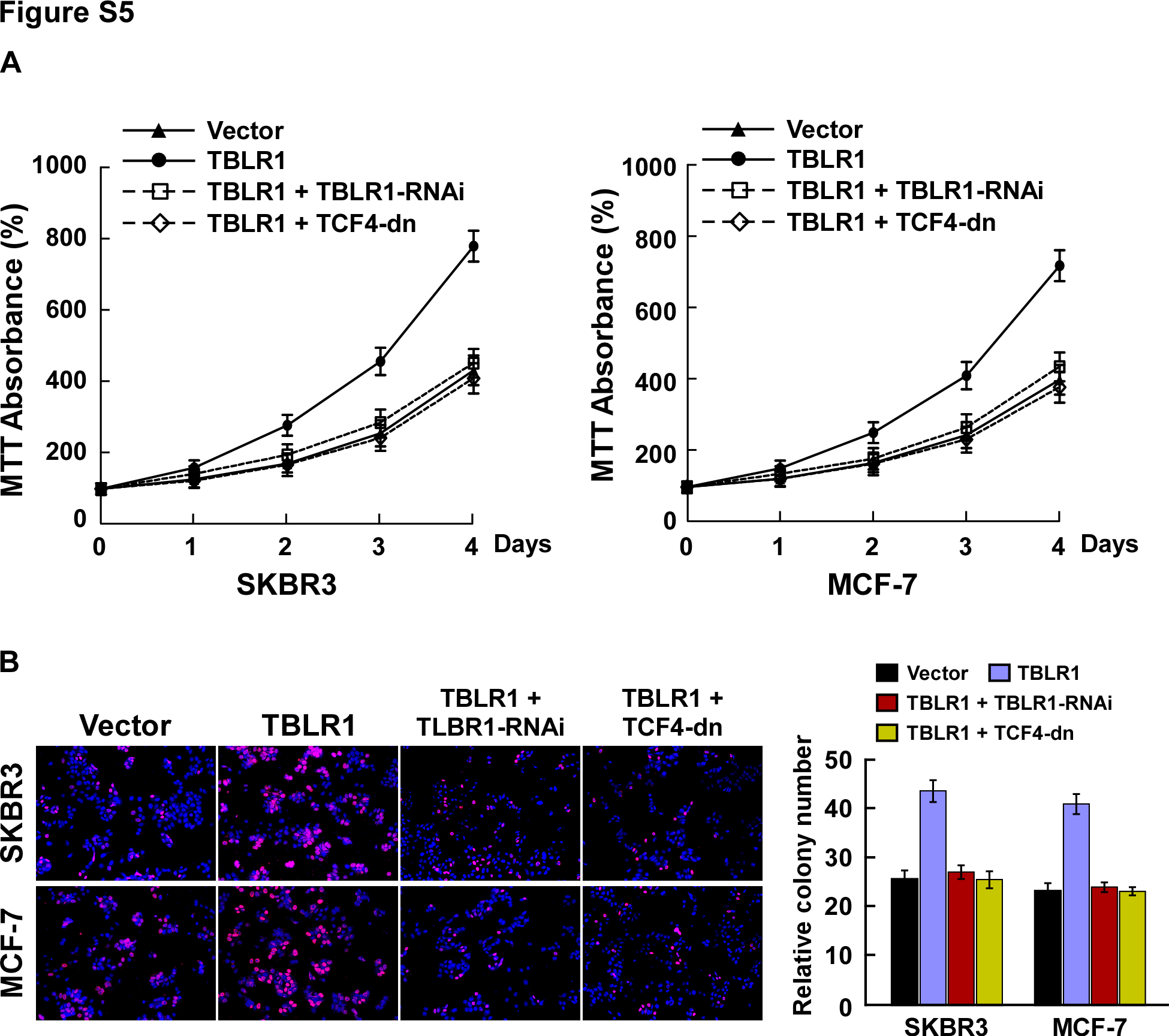

Supplement: Supplementary file 5 — Additional file 5: Figure S5.: Inhibition of Wnt/β-catenin signaling blocked the functional role of transducin (β)-like 1 X-linked receptor 1 (TBLR1). (A) MTT assays showed the proliferation rate of indicated cells. (B) Representative micrographs (left) and quantification of 5-bromodeoxy uridine (BrdU)-positive cells. Error bars represents the mean ± SD of three independent experiments; *P <0.05. (TIFF 589 KB) [file 13058_2014_465_MOESM5_ESM.tiff]

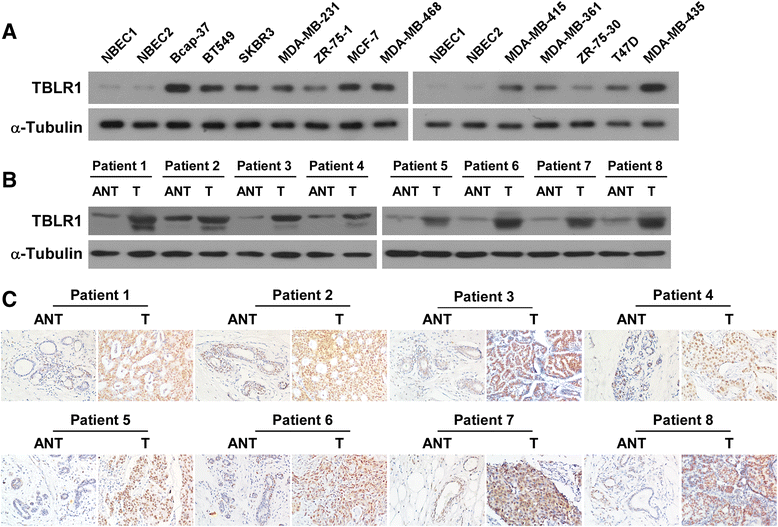

Supplement: Supplementary file 6 — Authors’ original file for figure 1 [file 13058_2014_465_MOESM6_ESM.gif]

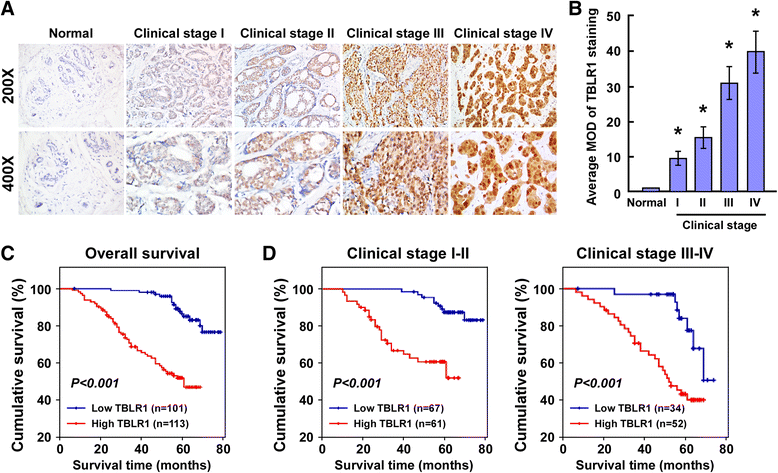

Supplement: Supplementary file 7 — Authors’ original file for figure 2 [file 13058_2014_465_MOESM7_ESM.gif]

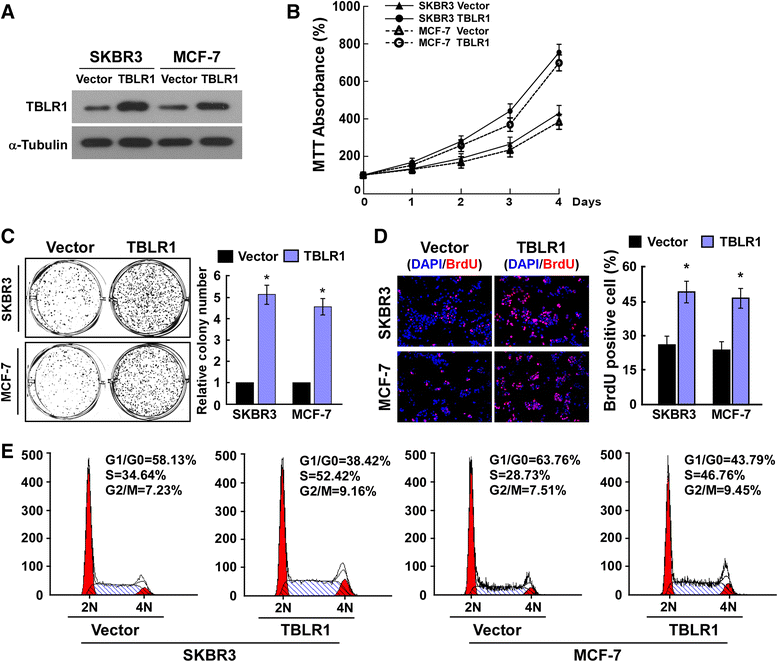

Supplement: Supplementary file 8 — Authors’ original file for figure 3 [file 13058_2014_465_MOESM8_ESM.gif]

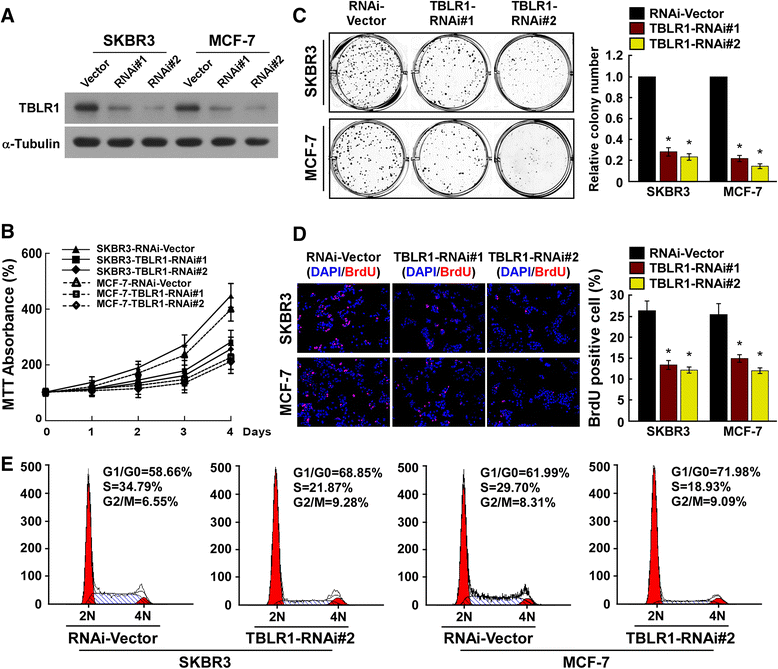

Supplement: Supplementary file 9 — Authors’ original file for figure 4 [file 13058_2014_465_MOESM9_ESM.gif]

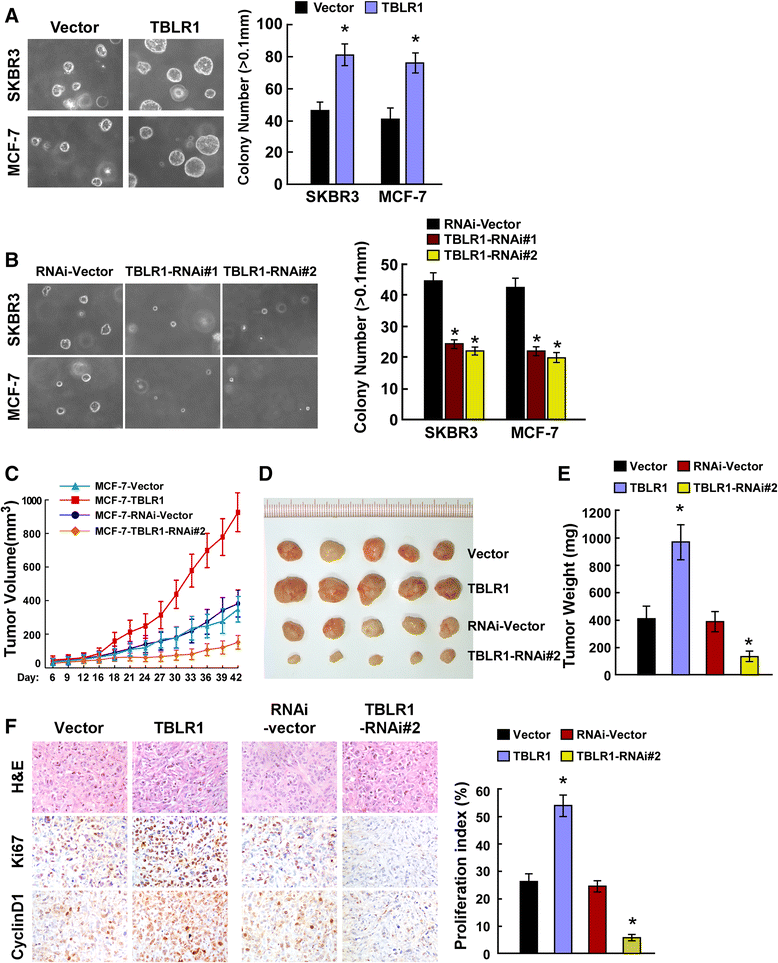

Supplement: Supplementary file 10 — Authors’ original file for figure 5 [file 13058_2014_465_MOESM10_ESM.gif]

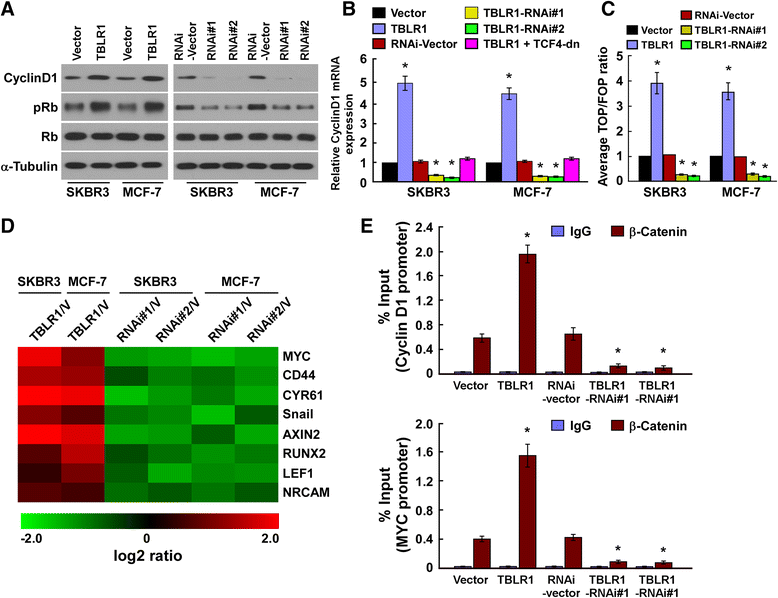

Supplement: Supplementary file 11 — Authors’ original file for figure 6 [file 13058_2014_465_MOESM11_ESM.gif]

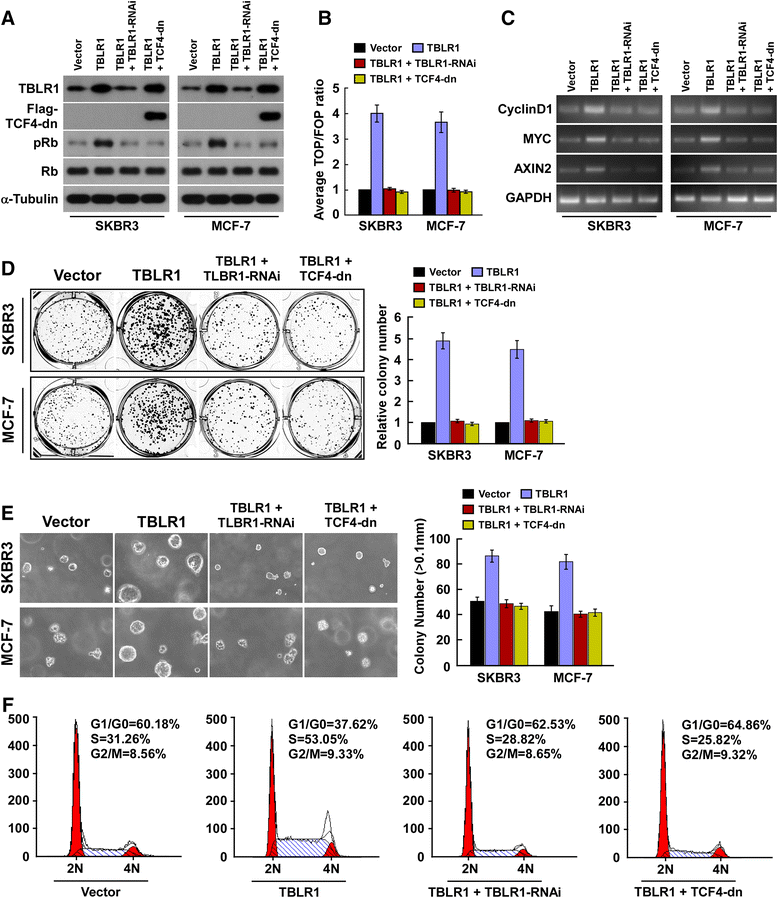

Supplement: Supplementary file 12 — Authors’ original file for figure 7 [file 13058_2014_465_MOESM12_ESM.gif]

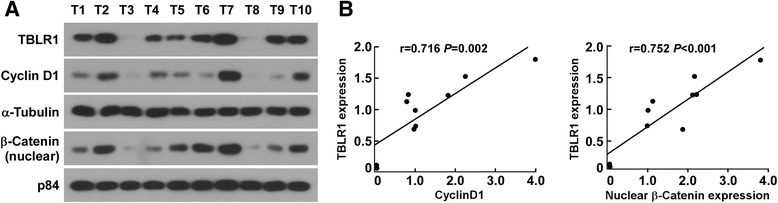

Supplement: Supplementary file 13 — Authors’ original file for figure 8 [file 13058_2014_465_MOESM13_ESM.gif]
